# Restoration and functional analysis of the SGI1 resolution system – SGI1 multimers are eliminated by the reactivated resolution

**DOI:** 10.1038/s41598-025-06025-6

**Published:** 2025-07-01

**Authors:** Alexandra Veress, Mónika Szabó, János Kiss

**Affiliations:** 1 Department of Microbiology and Applied Biotechnology, Institute of Genetics and Biotechnology, Hungarian University of Agriculture and Life Sciences, Gödöllő, Hungary; 2 Agribiotechnology and Precision Breeding for Food Security National Laboratory, Gödöllő, Hungary

**Keywords:** Bacterial evolution, Bacterial genetics, Microbial genetics, DNA recombination

## Abstract

SGI1 and the related elements that are specifically mobilized by the IncA- and IncC-family plasmids are efficient agents in the dissemination of multi-resistance in Gammaproteobacteria. The In104 gene cluster responsible for multi-resistance in these genomic islands is generally integrated into the conserved SGI1 backbone, upstream of a resolvase gene, presumably by *res*-hunting transposition events. In this work we demonstrate that precise deletion of In104 cluster with one copy of its flanking direct repeats restores the *res* site belonging to the resolvase gene, leading to an active Tn*3*-like resolution system. The entire *res* site and its subsites have been identified and the resolvase activity has been demonstrated in plasmid-based recombination assays. The major effect of the reactivated resolution system seems to be the rapid elimination of SGI1 multimers in SGI1 transconjugants. It has been shown that wt SGI1-C and the resolvase-deleted SGI1ΔIn104 variant produce significantly more concatemers, which persist for longer periods in transconjugants, than SGI1ΔIn104 with a functional resolution system. High prevalence of inactivated *res* systems among the multidrug-resistant members of SGI1-family suggests that the ability to produce more and more stable multimers in SGI1 transconjugants may confer evolutionary advantage to these elements.

**Supplementary Information:**

The online version contains supplementary material available at 10.1038/s41598-025-06025-6.

## Introduction

*Salmonella* Genomic Island 1 (SGI1) variants and related elements (SGI1-REs) form a large group of integrative mobilizable genomic islands that have an important role in the dissemination of multidrug resistance phenotype especially in Enterobacterales. Since the discovery of SGI1^1^ in multidrug-resistant (MDR) clones of *Salmonella* Typhimurium DT104, a pandemic clone detected in the early 1980s that became predominant worldwide in the early 1990s^2^, 37 variants and more distant relatives have been characterized. Recently 89 new members of SGI1 family occurring in seven orders of Gammaproteobacteria have been found in the NCBI databases^3^. SGI1-REs^4^ classified into 7 clusters based on their integrase gene^3^ generally integrate at the 3’ end of the tRNA-modification GTPase gene *trmE* (formerly known as *thdF* or *mnmE*)^5^, however, three exceptions were also found where the insertion occurred between *sodB* and *purR*. SGI1-like elements^6,7^ share a conserved backbone consisting 26–28 ORFs, which in most cases is interrupted by a more variable gene cluster responsible for the MDR phenotype. The most conserved part of the backbone includes an integration/excision (*int* and *xis*) module^8^; a replication module consisting of *oriV* and a two-gene operon encoding a regulatory leader peptide (S004) and *repA*^9^; genes for type IV secretion system (T4SS) components (*traN*_*S*_, *traG*_*S*_, *traH*_*S*_)^8,10^; an operon encoding FlhDC-family regulators^11^; a mobilization module, including the transfer origin (*oriT*) and genes for two mobilization proteins MpsA and MpsB, and a small RNA, sgm-sRNA^12,13^; and several small ORFs with as yet unknown functions (S008-S010, S013-S018, S021-S022), or with roles in incompatibility with IncC plasmids (*sci*)^14^. For most elements, the region downstream of *oriT* contains a helicase and a nuclease gene^8^; a toxin-antitoxin (TA) system^15^; a resolvase gene (*res*); and the conserved ORF *S044*^8^. However, some SGI1 variants lack the helicase and nuclease gene, and the TA system is replaced by a restriction modification system in some SGI1-REs like *Acinetobacter* Genomic Island 1 (AGI1)^16^, several variants of *Proteus* Genomic Island 1 (PGI1) and members of the newly defined Cluster 5, which comprises SGI1-REs from *Vibrio* and *Shewanella* species. Additionally, an unrelated TA system was found in the SGI1-RE of *V. cholerae* VN-2808, and the *res* gene is missing or replaced by a distinct one in some elements belonging to different clusters^3^.

SGI1-RE genes for excision, replication and T4SS components are under the control of promoters regulated by the FlhDC-family activator AcaCD of IncC plasmids and possibly by the self-encoded activator SgaDC^9–11,17,18^indicating that these elements can be mobilized by IncC (and probably by the closely related IncA) plasmids as has already been demonstrated for SGI1 ^11,19,20^ PGI1^21^, PGI2^22^ and AGI1^23^. SGI1 is not simply mobilized by IncC plasmids, but a complex parasitism-like interaction between the island and the plasmid is established at the helper’s expense, leading to more efficient transfer and stabilization of the island and the rapid destabilization and loss of the plasmid^9–11,14,18,24^.

From a health perspective the most important part of SGI1 is the MDR region containing the antibiotic resistance (AR) genes, which poses a serious threat in both human and veterinary medicine. The MDR region of SGI1-REs is an In4-type In104 integron cluster^25^, which comprises two integrons with AR cassettes, integron-independent AR genes (*floR*, *tetRA*(G)), transposable elements (CR3, IS*6100*), and some additional genes of unknown function in the prototype SGI1^8^. As In4-like integrons, the In104 cluster is also flanked by 25-bp imperfect inverse repeats that are identical to the IRi/IRt ends of Tn*402*/Tn*5090*, a presumed ancestor of In4-like integrons^26^. Most SGI1 variants only differ in the resistance cassette content of integrons or display IS-mediated rearrangements within the In104 cluster, however, some SGI1-relatives such as PGI1, PGI2 and AGI1, show higher sequence divergence even in their backbones^3,4,7,22^. A vast majority of known SGI1-REs, identified mainly in pathogenic human or veterinary isolates, contain an MDR region, which is located almost exclusively upstream of the *res* gene. The only exception is SGI2 found in several *Salmonella* Emek and Virchow strains, where the In104 cluster is inserted into the helicase gene (S023)^27,28^. A few SGI1-REs, however, lacking an MDR region (SGI0, VGI) have previously been described^29,30 ^and a recent work identified several other MDR-free SGI1 relatives predominantly in *Vibrio*, *Shewanella* and marine bacteria^3^. The In104 cluster in SGI1-REs is generally surrounded by a 5-bp direct repeat (DR), suggesting its transpositional acquisition. Since Tn*402*-family transposons have been reported to target the resolution sites (*res* site) of resolution systems (RS) found in Tn*3*-family transposons or plasmids (known as *res* site hunter Tns)^26,31–34 ^it was assumed that In104 may also be integrated into a putative *res* site that belongs to the *res* gene of SGI1^25^. Although In104 generally lacks Tn*402* genes required for transposition (*tniABQ*) (except in SGI2-variants found in *S. enterica* Emek and Virchow, where *tniA* and a partial *tniB* bracketed by IRt copies are inserted in the 3’ end of In104^28,35^) and is therefore defective in autonomous transposition, movement of similar integrons triggered by transposases of a related Tn acting *in trans* has been documented^36,37^.

Resolvase enzymes together with their cognate recombination sites constitute site-specific recombination systems to resolve chromosome- and plasmid-dimers or cointegrates formed by homologous recombination or replicative transposition of Tn*3*-family transposons^38,39^. Both Ser- and Tyr-recombinases can occur in resolution modules and both types of enzymes mediate recombination between short (~ 28 to 30-bp) DNA motifs typically containing inversely repeated binding sites for the enzyme. Tn*3*-family resolvases (Res) are generally small (~ 180 to 210 aa) Ser-recombinases, however, several transposons encode larger enzymes (~ 310 aa) with a ~ 110 aa C-terminal extension. The *res* site of Tn*3*-like transposons is located upstream of the *res* gene, is ~ 120-bp long, and exhibits a conserved organisation of three subsites (boxI, II and III). Each subsite shows more or less dyad symmetry due to the presence of 12-bp inversely oriented Res binding sites. The distance between the central bases of the core site I and the accessory site II is generally 5 helical turns, but it can vary from 4 to 7 turns, however helical phasing is important for proper establishment of the synaptosome complex. Ser-recombinase resolvases cleave all four DNA strands at the central 2 bp of both core subsite I, while interaction with subsites II and III promotes the synapsis of resolvase bound to subsite I. All subsites are essential for recombination, however Res show different binding affinity to them. The strict topology of the synaptosome guarantees unidirectional recombination (i.e. resolution of cointegrates carrying two directly oriented *res* sites rather than recombination between two plasmids with a single *res* site), however, under special circumstances, cointegrates can also be formed^40,41^.

The role and activity of RSs of SGI1-REs have been poorly investigated to date. The resolvases appear to be best related to those of Tn*3*-family transposons, however their functionality and roles in spreading or maintenance of SGI1 is unknown. The *res* site in SGI1-REs, lacking the MDR region, and in SGI2, carrying In104 integrated into S023, may be intact, but its exact location and subsites have not yet been identified. Thus, the RS may be functional in these SGI1-REs, although its activity and role have not yet been analysed. On the other hand, the *res* site is likely to be disrupted in the majority of SGI1-REs, in which In104 is inserted upstream of the *res* gene, probably resulting in the inactivation of the RS.

In SGI1 and its derivatives like SGI1-C, In104 is integrated 71 bp upstream of the start codon of the *res* gene and thus, it probably disrupts the *res* site. To restore the presumed *res* site, the In104 cluster has been removed along with one copy of its flanking 5-bp DRs from the SGI1-C variant. In this work, we have shown that In104 deletion reactivates the RS. The Res–*res* site interaction has been demonstrated, the subsites identified, and the function of the RS examined in plasmid-based systems and on SGI1 derivatives. The effects of the active and inactive RSs on SGI1 transfer and multimer formation have also been analysed.

## Results

### Restoration of the resolution system of SGI1

**Fig. 1:**
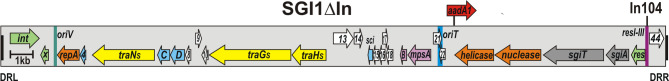
Schematic map of SGI1ΔIn. SGI1ΔIn contains the entire conserved SGI1 backbone with an Sm^R^/Sp^R^ cassette (*aadA1*) additionally inserted downstream of the helicase gene S023, but lacks the In104 cluster. Annotated ORFs S001-S044 are indicated by colour-coded arrows: green – recombination; orange – replication; blue – transcription regulator; yellow – T4SS components; purple – conjugation initiation, grey – TA system; red – antibiotic resistance gene; white – ORF of unknown function. Genes encoding hypothetical proteins are numbered according to the original numbering of SGI1 ORFs (e.g. ‘8’ refers to S008, etc.). Names of genes with known functions or identified homologs are marked accordingly with the following abbreviations: *x* – *xis*, *C* and *D* – *sgaC* and *sgaD*, *B* – *mpsB*. Colour-coded vertical lines indicate the relevant DNA elements: black – terminal direct repeats (DRL and DRR), green – origin of replication (*oriV*), blue – origin of transfer (*oriT*), purple – recombination (*res*) site obtained by the precise deletion of In104 with one copy of its flanking 5-bp DRs. The map is drawn to scale.

As the *res* gene (S027) of SGI1 seems to be intact, and it can be assumed that In104 was integrated into the *res* site of the primarily active RS of an ‘ancient’ SGI1 element by a transposition event, reactivation of the RS probably requires only the restoration of the original *res* site. It was carried out by precisely removing In104 together with a copy of the flanking DRs from SGI1-C in three steps using a scarless deletion method^42^. First, In104 cluster was replaced by a *cat* gene cassette conferring Cm^R^ phenotype. Since SGI1-C carries all AR genes in In104, a resistance marker gene had to be integrated elsewhere in the backbone before removing the Cm^R^ cassette. The *aadA1* gene from R100.1 plasmid^43 ^conferring Sm^R^/Sp^R^ and related closely to *aadA2* that was originally present in the In104 of SGI1-C, was inserted between the promoter region of sgm-sRNA and the 3’-end of the helicase gene (between P_S022_ and S023). It was previously shown that small deletions in this region caused no detectable changes in SGI1 functions^12,13 ^therefore insertion of an AR gene at this location should be neutral. After that, the putative *res* site was restored by eliminating the Cm^R^ gene. Since the resulting SGI1ΔIn104 (abbreviated as SGI1ΔIn – Fig. 1) variant and the wt SGI1-C are significantly different in size and gene content, the coding sequence of *res* gene between the start and stop codons was deleted in SGI1ΔIn to create the SGI1ΔInΔ*res* mutant, an isogenic but definitely resolution-deficient control variant.

### Confirming the binding activity of the res protein to the restored *res* site and its subsites

To obtain initial evidence on the functionality of the *res* site of SGI1ΔIn, N-terminally His-tagged resolvase protein was purified (Fig. S1) and used in EMSA with 5’FAM-labelled 207-bp probe representing the entire intergenic region between *res* and S044 genes of SGI1ΔIn. Gradually increasing the Res concentration, first only one, then two and finally three complexes were detected. (Fig. S2). This was consistent with the general structure of three-subsite-containing *res* sites of RSs of Tn*3*-family transposons.

**Fig. 2:**
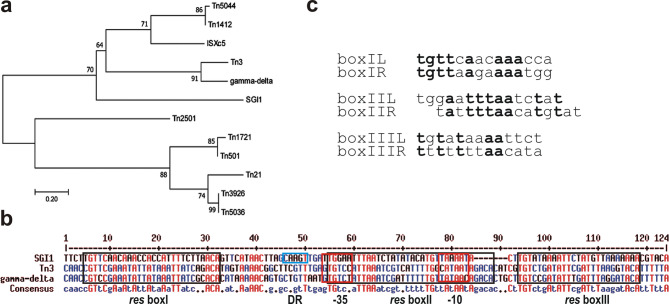
Prediction of subsites within the restored *res* site of SGI1. (**a**) Comparison of the restored *res* site of SGI1 with eleven *res* sites of Tn*3*, γδ, Tn*5036*, Tn*5044*, Tn*412*, IS*Xc5*, Tn*2501*, Tn*1721*, Tn*21*, Tn*501* and Tn*3926* transposons. The phylogenetic tree was generated by Maximum likelihood method, bootstrapping was repeated 500 times. (**b**) Specification of boxI-boxIII based on the alignment with the most similar *res* sites of Tn*3* and γδ transposons. Conserved subsites, the 5 bp that has been duplicated upon In104 insertion in wt SGI1 and the −35 and −10 promoter boxes of the *res* gene are boxed by black, blue and red, respectively. (**c**) Symmetry of the predicted *res* boxes. The reverse complementer sequence of the right (R) halves are aligned with the left (L) halves of the *res* boxes. Symmetric positions are highlighted in bold.

In order to localize the *res* site in the intergenic region and to identify its subsites, *res* sites and the resolvase proteins of several transposon-derived RSs were compared to those of SGI1ΔIn. The phylogenetic tree generated for 13 *res* sites (Tn*3*^44^; γδ^45^; Tn*5036*, Tn*5044*, Tn*412*^31^; IS*Xc5*^46^; Tn*2501*^47^; Tn*1721*, Tn*21*^48^; Tn*501*^49^; Tn*3926*^50^ and that of SGI1ΔIn) revealed that the closest relatives of SGI1 *res* site form two clusters, one with Tn*3* and γδ, and the other with Tn*5044*, Tn*412* and IS*Xc5 res* sites (Fig. 2a). Resolvases of these elements also appeared to be the closest relatives of SGI1 Res, which was shown up on the phylogenetic tree as a sister group of the Tn*5044*, Tn*412*, IS*Xc5* cluster (Fig. S3). Comparison of the restored SGI1 *res* site with its five closest relatives (Fig. S4a) or separately with those in the two clusters (Fig. S4b,c) indicated that the best alignment can be obtained with *res* sites of Tn*3* and γδ transposons. Therefore, they were used to predict the entire *res* site and its subsites in SGI1 (Fig. 2b). Alignment with the well-defined Tn*3* and γδ *res* sites showed that boxI and boxIII of SGI1 *res* site are more similar to the corresponding subsites (46% and 48%, respectively), than boxII that is less conserved (35%) (Fig. 2c). Based on this prediction, the full SGI1ΔIn *res* site was defined as a 107-bp sequence including three subsites with the following structure: boxI (28 bp) is separated from boxII (30 bp) by a 22-bp spacer region including the 5 bp that has been duplicated upon In104 insertion in wt SGI1-C, while boxII and boxIII (25 bp) are separated by a 2-bp spacer (Fig. 2b).

**Fig. 3:**
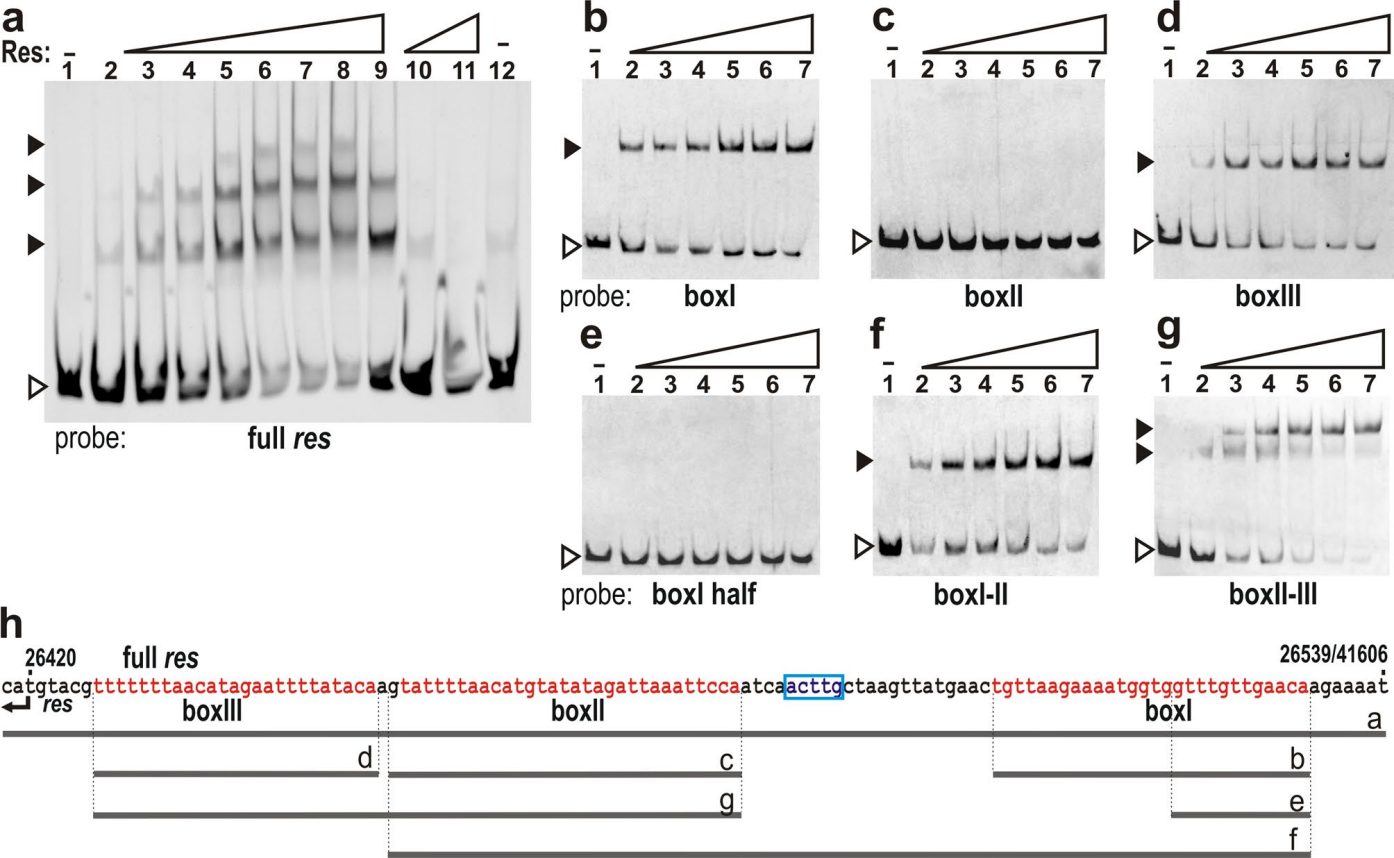
EMSA analysis of the entire *res* site and its predicted subsites (boxI, II and III). (**a**) Detection of DNA-protein complexes formed upon His-tagged SGI1 resolvase (Res) binding to the full *res* site. All binding reactions contained 5.6 ng 5’FAM-labelled 122-bp probe representing the predicted *res* site in SGI1ΔIn. Lanes 1–9 – increasing amount of purified Res: 0, 0.075, 0.225, 0.45, 0.9, 1.35, 1.8, 2.25, 2.7 µg, Lanes 10–11 – 1.2 and 1.5 µg Res and 1.7 µg (~ 300-fold) unlabelled probe that was added to the binding reaction 10 min prior to the labelled probe, Lane 12 – 2.7 µg protein preparate obtained from BL21 (DE3)/pET16b strain with no Res protein (negative control). Open arrowhead points to unbound probe, numbered filled arrowheads indicate shifted complexes. (**b**) Res binding to boxI. (**c**) Res binding to boxII. (**d**) Res binding to boxIII. (**e**) Res binding to the half of boxI. (**f**) Res binding to the boxI-boxII region of *res* site. (**g**) Res binding to the boxII-boxIII region of *res* site. In panels b-g, all reactions contained 8.0 ng of 5’FAM-labelled probes (see panel h) and increasing amounts of Res protein as follows: Lanes 1–7 – 0, 0.7, 1.4, 2.1, 2.8, 3.5 and 4.9 µg. Open arrowheads point to the unbound probe, filled arrowheads indicate the shifted DNA-protein complexes. (**h**) Sequence of the restored *res* site. The predicted boxes are highlighted in red, coordinates refer to base positions in SGI1ΔIn/wt SGI1starting with the first nucleotide of wt SGI1 DRL. Start codon of the *res* gene and the 5 bp that have been duplicated upon In104 insertion in wt SGI1 are indicated. Horizontal lines (**a**–**g**) represent the sequence included in the 5’FAM-labelled probes used in EMSAs on panels a-g (see also Suppl. Table S4). Original gels are presented in (Supplementary Fig. S7).

To prove that the Res protein binds to the predicted full *res* site, the amplicon containing the three subsites (boxI-III) without substantial flanking sequences was 5’FAM-labelled and used in EMSA. Similarly to the probe representing the entire *res*-S044 intergenic region, all three complexes could be detected using the highest protein concentrations. The shifted bands could be eliminated by adding the same but unlabelled DNA fragment to the reaction prior to the labelled probe, demonstrating the binding specificity (Fig. 3a,h). Formation of the expected three complexes confirmed our prediction for the full *res* site. To verify the binding of Res to each predicted subsite, boxI, boxII and boxIII were individually analysed in EMSAs. While boxI and boxIII gave one shifted band, the Res protein did not bind to boxII under the same conditions, nor did to half of the boxI sequence (Fig. 3b–e,h). These results verified that the prediction was correct for boxI and boxIII and indicated that a half binding site is not sufficient for Res binding. However, shifting of boxII failed, suggesting that its binding is cooperative.

For further analysis of Res–boxII interactions, two additional probes containing boxII with boxI and boxII with boxIII along with the respective spacing sequences were applied. The boxI–22-bp spacer–boxII (boxI-II) probe gave only one shifted band, similarly to the boxI probe, while boxII–2-bp spacer–boxIII (boxII-III) probe resulted in two shifted bands (Fig. 3f–h), indicating that Res binding to boxII depends on the Res–boxIII interaction. Although this approach is not as accurate for binding site determination as footprinting techniques, predictions of boxes based on those of Tn*3* and γδ and the subsequent EMSA assays convincingly identified the restored *res* site of SGI1ΔIn.

### Functional analysis of the putative resolution system of SGI1 – recombination by the Res protein in vivo and in vitro

**Fig. 4:**
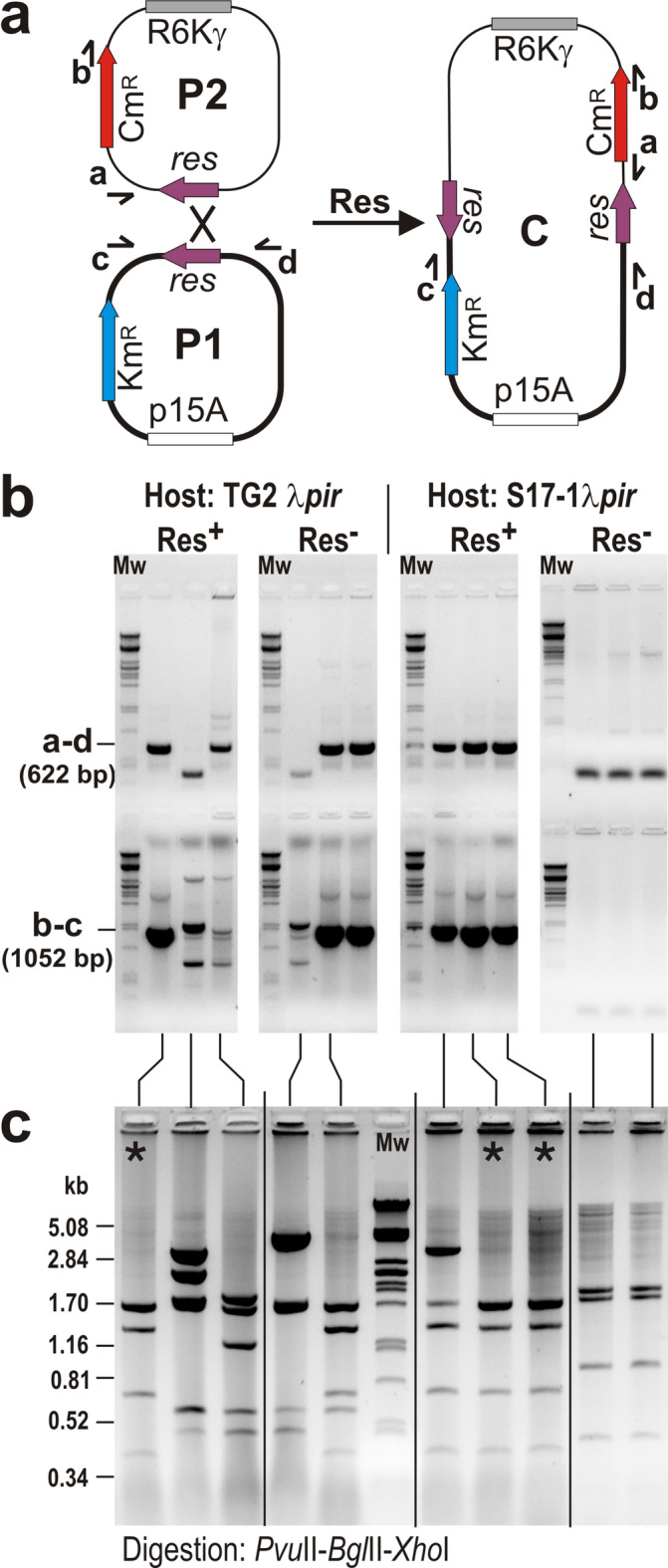
Cointegrate formation promoted by SGI1 Res. (**a**) Schematic maps of the *res*-site-containing parental plasmids (P1 = pJKI1088 and P2 = pJKI1092) and the expected structure of their cointegrate (C = pJKI1099) formed via site-specific recombination between *res* sites. Replication origins are indicated by white (p15A) and grey (R6Kγ) rectangles, resistance markers by blue (Km) and red (Cm) arrows, and the restored *res* sites by purple arrows. Direction of oligonucleotide primers used for amplification of junctions in the cointegrate are shown by small arrows marked with lower case: a – FRTfor, b – cat3.2, c – pucrev25, d – pBRBgl. (**b**) A representative set of colony PCR results from Km^R^Cm^R^Ap^S^ TG1 transformants obtained from transformation of plasmid DNA isolated from TG2 λ*pir* and S17-1 λ*pir* hosts, enabling replication of both parental plasmids and the resolvase-producing (Res^+^, pAVE20) or non-producing (Res^−^, pKK223-3) control plasmids. The TG1 host does not support replication of R6Kγ-based parental plasmid P2, thus TG1 transformants selected for the resistance markers of both parental plasmids (Km^R^Cm^R^) should be cointegrates. Resolvase-producing (Res^+^) or non-producing (Res^−^) TG2 λ*pir* and S17-1 λ*pir* indicate the origin of the plasmid DNA introduced into the TG1 host. The junction regions of cointegrates were amplified using primer pairs a-d and b-c. The expected sizes of amplicons specific for the correct junctions are indicated. (**c**) Restriction mapping of cointegrates isolated from Km^R^Cm^R^ TG1 transformants that showed different patterns of amplicons in colony PCR screening. Correct cointegrate structures are marked by asterisks. Mw – molecular-weight marker (λ DNA digested with *Pst*I) used in all gels. Original gels are presented in (Supplementary Fig. S8).

To investigate whether Res can catalyse recombination between two restored *res* sites, plasmid-based systems were developed where recombination was easily detectable. At first, the reverse reaction of resolution, i.e. the production of a cointegrate from *res*-site-bearing parental plasmids, was examined with the further aim of obtaining a regular cointegrate for subsequent experiments. The following three-plasmid system was established: two *res*-site-containing plasmids with different resistance markers and replication origins (Km^R^ –p15A and Cm^R^ –R6Kγ), but without extensive homology, were constructed (Fig. 4a). The R6Kγ plasmid can be maintained only in *pir*^*+*^ hosts. The third one was the Res protein producer (Res^+^) or the non-producer (Res^−^) empty vector (negative control), both were compatible with the others (Ap^R^ – ColE1). Although RSs have been evolved to carry out intramolecular recombination between directly oriented *res* sites located on the same DNA, the reversed process, i.e. cointegrate formation can also occur with low-frequency^40,41^. Therefore, the three plasmids were introduced in four parallel transformations into the *recA* strains TG2 λ*pir* and S17-1 λ*pir*, which allowed the formation of cointegrates via resolvase-mediated site-specific recombination. Then, plasmid DNA was isolated from Km^R^Cm^R^Ap^R^ colonies and transformed into a TG1 host that did not support the replication of an R6Kγ plasmid, thus, Cm^R^ phenotype could only occur when the resulting transformants carried a cointegrate of the *res*-site-containing parental plasmids, conferring Km^R^Cm^R^ phenotype. Transformants were titrated on LB + Km (selective for the p15A replicon) and LB + Km + Cm (selective for cointegrates) plates and the cointegrate frequency was expressed as their ratio (Table 1). A relatively high cointegration frequency was observed in the presence of the Res^+^ plasmid compared to the negative control in TG2 λ*pir*, whereas no significant difference was detected in S17-1 λ*pir*. However, colony PCRs specific for junctions of regular cointegrates (formed via *res* site × *res* site recombination) indicated that most Km^R^Cm^R^ TG1 colonies did not contain the correct structure (Fig. 4b). Plasmid DNA was isolated from colonies that gave different amplicons in colony PCR and analysed by restriction profiling. As expected, regular cointegrates were only obtained from colonies where both junctions could be amplified, but in some cases incorrect structures were obtained, even if the PCR results were correct (Fig. 4c, lanes 3, 6, 8). The frequency of Res-mediated cointegrate formation was then estimated based on the prevalence of correct cointegrates among the samples analysed, which was approx. 1.4 × 10^− 3^ in TG2 λ*pir* and 2.1 × 10^− 4^ in S17-1 λ*pir* (Table 1). Although many Km^R^Cm^R^ transformants were obtained from the Res^−^ negative controls from both hosts, none of them carried true cointegrates. This indicated that the relatively few correct cointegrates obtained in the presence of the Res-producing plasmid were products of the Res-mediated site-specific recombination. Junction regions of several correct cointegrates were confirmed by sequencing and one of them (named as pJKI1099) was used in subsequent resolution assays.

**Table 1:** Cointegrate formation in vivo.

host	Pproducer plasmid^a^	Km^*R*^ TG1 transformant titer^b^	Km^*R*^Cm^*R*^ TG1 transformant titer^b^	Frequency of cointegrates (Km^*R*^Cm^*R*^/Km^*R*^)	Correct cointegrates per Km^*R*^Cm^*R*^ colonies analysed	Estimated frequency of Res-mediated cointegrates
TG2 λ*pir*	R+	2.3 ± 1.1 × 10^4^	7.2 ± 5.7 × 10^2^	2.7 ± 1.7 × 10^− 2^	1/20	~ 1.4 × 10^− 3^
	R-	1.3 ± 0.1 × 10^5^	4.0 ± 1.5 × 10^1^	3.2 ± 1.3 × 10^− 4^	0/12	< 2.6 × 10^− 5^
S17-1 λ*pir*	R+	6.2 ± 1.2 × 10^4^	3.4 ± 2.2 × 10^1^	6.2 ± 4.5 × 10^− 4^	4/12	~ 2.1 × 10^− 4^
	R-	5.5 ± 2.6 × 10^4^	1.8 ± 0.8 × 10^1^	4.0 ± 2.3 × 10^− 4^	0/12	< 3.3 × 10^− 5^

^*a*^R+: pAVE20 (Res-producer, Res^+^), R^−^: pKK223-3 (negative control empty vector, Res^−^).

^*b*^Means are calculated from data of four parallel transformations.

**Fig. 5:**
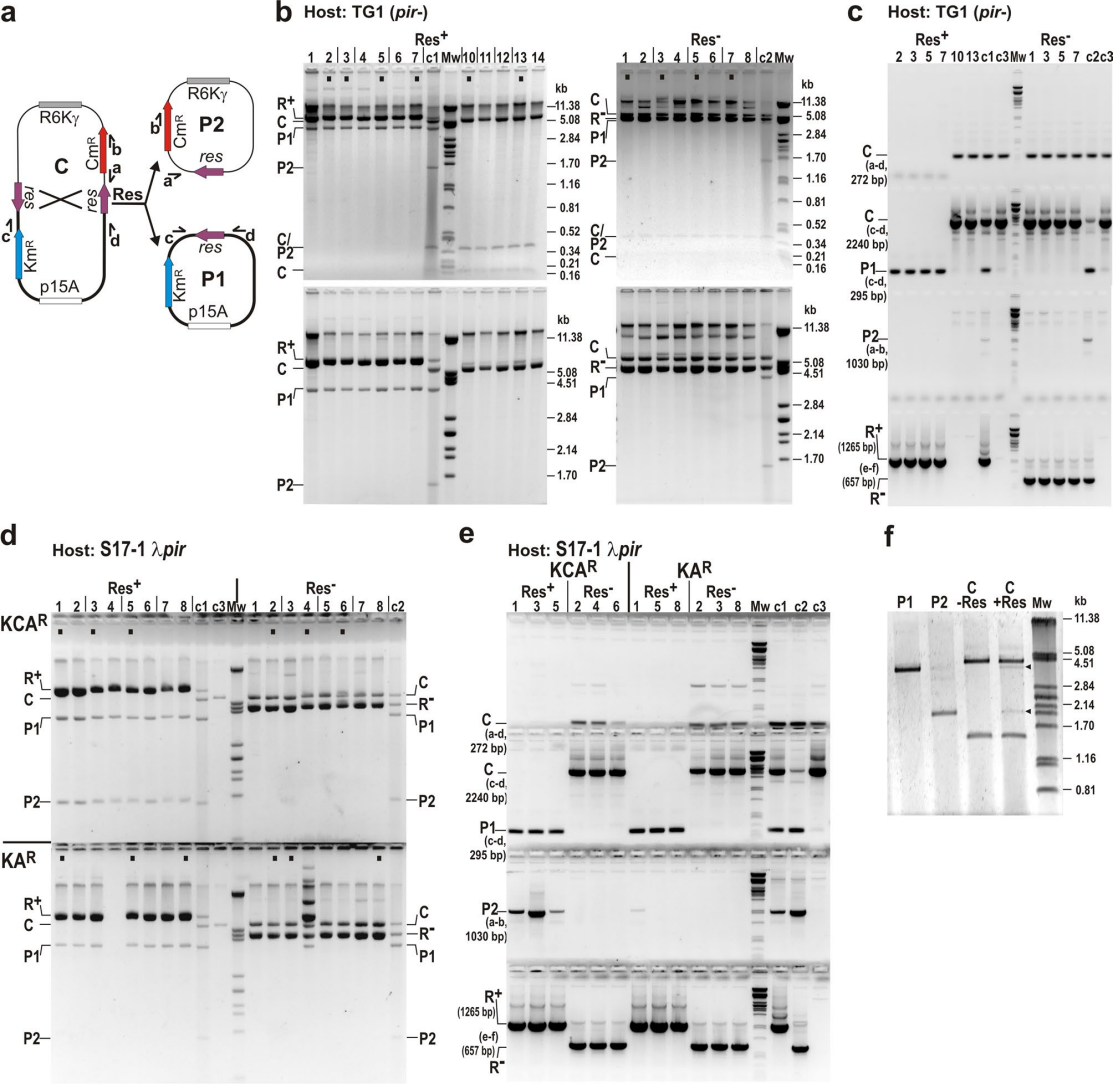
Detection of SGI1 Res-mediated cointegrate resolution. (**a**) Schematic maps of the cointegrate with directly oriented *res* sites (C = pJKI1099) and its parental plasmids (P1 = pJKI1088 and P2 = pJKI1092) formed via site-specific recombination between *res* sites. Symbols are as in (Fig. 4). Note that P2 is non-replicable in the TG1 host. Direction of oligonucleotide primers used for identification of different plasmid species are shown by small arrows marked with lower case: a – FRTfor, b – catNco, c – pucrev25, d – pucfor24. (**b**) Decay of the cointegrate in a *pir*^−^ host. Plasmid DNA was isolated from Km^R^Ap^R^ and Km^R^Cm^R^ colonies obtained from four parallel transformations of the Res-producer (Res^+^, pAVE20) or non-producer (Res^−^, pKK223-3) control plasmid into TG1 cells already harbouring the cointegrate pJKI1099. The TG1 strain (*pir*^−^) does not allow replication of the P2 (R6Kγ) replicon. Plasmid DNA was digested with *Hin*dIII and run on 1% agarose gel. Bottom panels show the same gels after ON run at 9 mA. Origin of fragments are indicated as follows: C – cointegrate, P1 and P2 – parental plasmids emerging via cointegrate resolution, R^+^ – Res-producer plasmid, R^−^ - non-producer negative control plasmid. Note that the 376-bp fragment can derive from both C and P2 plasmids (marked as C/P2). Samples deriving from different parallel transformations are separated by vertical lines. Res^+^ panel, Lanes 1–7 – representative set of plasmid DNA from Km^R^Ap^R^ Res^+^ transformants; Lane c1 – mixture of plasmid DNA of C, P1, P2 and Res^+^ (control 1); Lanes 10–14 – plasmid DNA from the Km^R^Cm^R^ secondary colonies; Res^−^ panel, Lanes 1–8 – a representative set of plasmid DNA from Km^R^Ap^R^ Res^−^ transformants; Lane c2 – mixture of plasmid DNA of C, P1, P2 and Res^−^ (control 2). DNA samples marked with dots were subjected to PCR tests shown in panel C. (**c**). PCR detection of plasmid species in the selected samples. Primer pairs specifically detect C, P1 and P2 are as in (Fig. 4), the Res-producer and control vectors (R^+^ and R^−^) were detected using primers e – pKKfor and f – rrnBrev2. Numbering of lanes refers to the dotted samples in panel b. The c1 and c2 controls are as in panel b, control c3 is the sole cointegrate. Primer pairs and the size of amplicons specific for each plasmid species are shown. Note that primer pair c-d amplifies a 295-bp fragment from P1 and a 2.24-kb fragment from C. (**d**) Decay of the cointegrate in a *pir*^+^ host. Plasmid DNA was isolated from Km^R^Cm^R^Ap^R^ and Km^R^Ap^R^ colonies obtained from four parallel transformations of the Res-producer (Res^+^, pAVE20) or non-producer (Res^−^, pKK223-3) plasmids into S17-1 λ*pir* cells harbouring the cointegrate pJKI1099. S17-1 λ*pir* allows replication of all the three plasmid types. Lanes 1–8 in Res^+^ and Res^−^ panels show representative sets of *Hind*III-digested plasmid DNA from resolvase-producing (Res^+^) and non-producing (Res^−^) S17-1 λ*pir* transformants, respectively. Upper part of the gel shows samples from transformants selected for Km^R^Cm^R^Ap^R^ (marked as KCA^R^), while the lower part shows those obtained from Km^R^Ap^R^ (KA^R^) selection. Plasmid samples were run on 1% agarose gel ON at 9 mA (small fragments derived from C and/or P2 plasmids run out of the gel). Symbols are as in panel b. DNA samples marked with dots were analysed by PCRs shown in panel e. (**e**) PCR detection of plasmid species in the selected samples dotted in panel D. Primer pairs, controls and symbols are as in panel b, c and d. (**f**) Cointegrate resolution in vitro. The cointegrate plasmid DNA was incubated with (C + Res) or without (C-Res) the purified His-tagged Res protein, then digested with *Sca*I, as well as control plasmids P1 and P2. Arrowheads point to P1 and P2 parental plasmids derived from cointegrate resolution. Original gels are presented in (Supplementary Fig. S9).

To test the default function, i.e. cointegrate resolution, of the RS, Res^+^ or Res^−^ plasmids were introduced into a TG1 host already harbouring the previously isolated Km^R^Cm^R^ cointegrate, pJKI1099. If the resolvase performs the expected recombination, the cointegrate decays into the parental plasmids. As one of them is *pir*-dependent and unable to replicate in the TG1 host, it is expected to be lost (Fig. 5a). To detect this, transformants were titrated on LB + Km + Ap and LB + Km + Cm + Ap plates and the resistance phenotype of individual colonies was monitored by replica plating. First indication for the activity of RS was that no Km^R^Cm^R^Ap^R^ transformants were obtained at all with the Res^+^ plasmid, while frequency of the two phenotype categories (Km^R^Ap^R^ and Km^R^Cm^R^Ap^R^) among transformants obtained with the Res^−^ control vector was almost the same (Table 2a). Furthermore, phenotype tests of individual colonies showed that none of Res + Km^R^Ap^R^ transformants preserved the Cm^R^ marker, while all Res^−^ control transformants did (Table 3). Interestingly, a few very small secondary colonies grew in the footprints of replica-plated Km^R^Ap^R^ Res^+^ colonies on LB + Km + Cm + Ap plates, whose subsequent phenotype test showed that they were Km^R^Cm^R^Ap^S^, suggesting the loss of Res-producer plasmid from some cells in the original colony before cointegrate resolution. Restriction and PCR analyses of the plasmid content of some transformants from resistance phenotype categories proved that the Km^R^Ap^R^Res^+^ colonies contained only the Res-producer plasmid and the Km^R^ p15A-based parental plasmid (P1), and lacked the cointegrate (i.e. resolution led to the loss of entire Cm^R^ R6Kγ-based parental plasmid), while Km^R^Ap^R^Res^−^ colonies preserved the intact cointegrate (C) along with the negative control vector (Fig. 5b,c). Plasmid content of the small secondary colonies proved to be original cointegrates without the Res-producer plasmid.

**Table 2:** Cointegrate resolution in vivo.

Host		Plasmids^a^	Total cell titer on LB^b^	Titer on LB + Km + Ap^b^	Frequency of Km^*R*^Ap^*R*^ transformants	Titer on LB + Km + Cm + Ap^b^	Frequency of Km^*R*^Cm^*R*^Ap^*R*^ transformants
**a**	TG1	C and R+	5.2 ± 0.5 × 10^8^	4.4 ± 0.5 × 10^4^	8.5 ± 0.8 × 10^− 5^	< 2.0 × 10^2 *c*^	< 3.9 × 10^− 7^
		C and R-	4.9 ± 0.6 × 10^8^	8.0 ± 2.7 × 10^4^	1.7 ± 0.6 × 10^− 4^	7.5 ± 2.6 × 10^4^	1.5 ± 0.4 × 10^− 4^
**b**	S17-1 λ*pir*	C and R+	2.9 ± 0.3 × 10^8^	3.2 ± 2.4 × 10^1^	1.2 ± 0.8 × 10^− 7^	2.2 ± 1.3 × 10^1^	6.2 ± 4.8 × 10^− 8^
		C and R-	4.9 ± 0.8 × 10^8^	5.4 ± 2.8 × 10^2^	1.0 ± 0.4 × 10^− 6^	7.0 ± 1.4 × 10^1^	1.5 ± 0.5 × 10^− 7^

^*a*^C: pJKI1099 (cointegrate), R^+^: pAVE20 (Res-producer, Res^+^), R^−^: pKK223-3 (negative control empty vector, Res^−^).

^*b*^Means are calculated from data of four parallel transformations.

^*c*^No colonies were obtained.

**Table 3:** Cointegrate resolution in TG1 host – replica plating.

Plasmids transformed^a^	Km^*R*^Ap^*R*^ transformant colonies analysed^b^		Frequency of intact cointegrates (%)
	Km^*R*^Ap^*R*^	Km^*R*^Cm^*R*^Ap^*R*^	
C and R^+^	509	0	< 0.19
C and R^−^	2242	2242	100

^*a*^C: pJKI1099 (cointegrate), R^+^: pAVE20 (Res-producer, Res^+^), R^−^: pKK223-3 (negative control empty vector, Res^−^).

^*b*^Data are from four parallel transformations.

Similar assay was carried out using S17-1 λ*pir* strain allowing the replication of all plasmids. In this case, comparable frequencies of Km^R^Ap^R^ and Km^R^Cm^R^Ap^R^ transformants were obtained regardless of the Res^+^ or Res^−^ producer plasmid, although the negative control resulted in slightly more transformants (Table 2b). Restriction profiling and PCR analyses of plasmid content of Km^R^Cm^R^Ap^R^ Res^+^ and Res^−^ transformants proved that Res^+^ colonies contained both parental (P1 and P2) and the Res-producer plasmids, but without the cointegrate, while Res^−^ colonies preserved the cointegrate and no parental plasmids were detected (KCA^R^ in Fig. 5d,e). In S17-1 λ*pir* host, both the cointegrate and the co-resident parental plasmids arising from cointegrate resolutin could confer Km^R^Cm^R^ phenotype. Therefore, plasmid DNA of the original Km^R^Cm^R^Ap^R^ transformants (marked with dots in Fig. 5d) was re-transformed into S17-1 λ*pir* to examine the efficiency of cointegrate resolution. Transformants were selected on LB + Km and LB + Cm plates, and the colonies were replica plated onto LB + Km + Cm plates to detect the remaining cointegrates. The Res^+^ samples provided only a few Km^R^Cm^R^ colonies, all containing exclusively the parental plasmids but not the cointegrate (these were co-transformants of P1 and P2), while the Res^−^ samples gave solely cointegrates. The results indicated that resolution efficiency was close to 100% (Table 4). Similar results were obtained from restricion analyses of plasmid DNAs obtained by re-transforming the plasmid content of the original Km^R^Ap^R^ Res^+^ and Res^−^ transformants into S17-1 λ*pir*: no cointegrates from Res^+^ and no parental plasmids from Res^−^ transformants were detected. The only difference was that the R6Kγ-based parental plasmid (P2) was mostly lost from Res^+^ transformants even though it is replicable in S17-1 λ*pir* host, indicating that this plasmid is unstable without Cm-selection in cells containing two other plasmids (p15A and ColE1, Km^R^Ap^R^ in Fig. 5d,e).

**Table 4:** Cointegrate resolution in S17-1 λ*pir* host – replica plating.

Plasmids transformed^a^	Km^*R*^ transformants analysed^b^		Frequency of intact cointegrates (%)	Cm^*R*^ transformants analysed^b^		Frequency of intact cointegrates (%)
	Km^*R*^	Km^*R*^Cm^*R*^		Cm^*R*^	Km^*R*^Cm^*R*^	
C and R^+^	1336	22 ^*c*^	< 7.5 × 10^− 2^	1146	6 ^*c*^	< 8.7 × 10^− 2^
C and R^−^	1178	1178	100	820	820	100

^*a*^C : pJKI1099 (cointegrate), R^+^: pAVE20 (Res-producer, Res^+^), R^−^: pKK223-3 (empty vector, Res^−^).

^*b*^Data are from three parallel transformations of DNA samples marked with dots in (Fig. 5d), Part KCA^R^.

^*c*^These Km^R^Cm^R^ transformants contained the parental plasmids P1 and P2 without cointegrate (P1 + P2 co-transformants).

Finally, the resolution was also examined in an in vitro reaction, where the cointegrate DNA was incubated with the purified His-tagged Res protein, then digested by *Sca*I and analysed on SybrGreen-stained agarose gel. *Sca*I linearizes both parental plasmids, but cuts the cointegrate into two distinct fragments. Although the resolution efficiency was lower than in vivo, both parental plasmids appeared in an approximately equal amount in the Res-treated sample (Fig. 5f), together representing ~ 11% of the total amount of plasmid DNA (estimation is based on band intensities). Thus, the His-tagged Res protein proved to be active in catalysing recombination in vitro. The in vivo and in vitro results demonstrated that the restored *res* site and the SGI1-encoded resolvase constitute a functional and highly active resolution system.

### Analysis of effects of the restored resolution system on SGI1 transconjugants

**Fig. 6:**
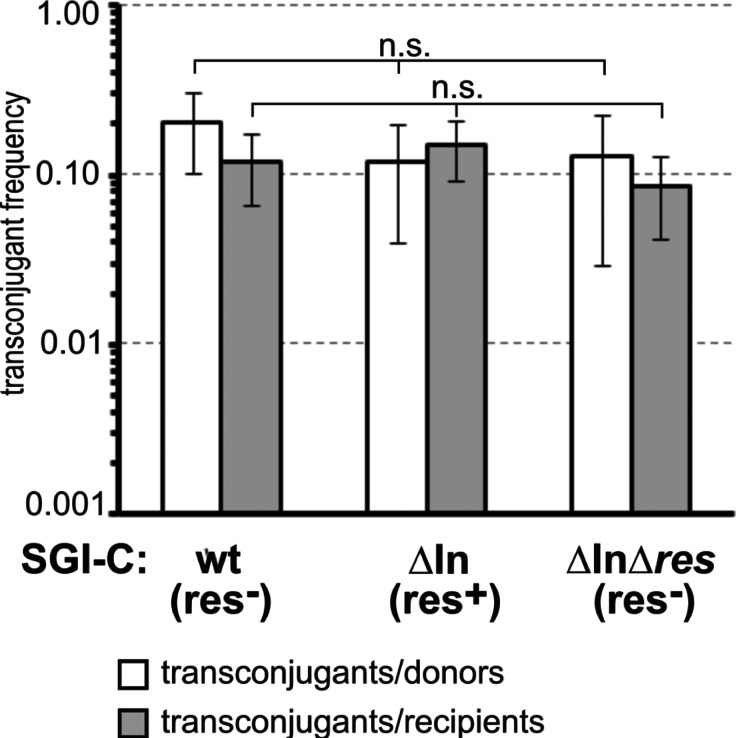
Comparison of transfer frequencies of the wt, ΔIn and ΔInΔ*res* SGI1-C variants. *n.s.* not significant (*P* > 0.05). P values of T-tests comparing the data of ΔIn and ΔInΔ*res* to that of wt: transconjugants/donors – wt/ΔIn *P* = 0.104, wt/ΔInΔ*res* *P* = 0.174; transconjugants/recipients – wt/ΔIn *P* = 0.315, wt/ΔInΔ*res* *P* = 0.237.

To examine the effects of the re-activited RS on SGI1 transfer, wt (resolution deficient, res^−^) and the isogenic ΔIn (resolution competent, res^+^) and ΔInΔ*res* (res^−^) mutant SGI1-C were compared in a standard mobilization assay where the Type 2 IncC plasmid R55 was used as helper. Although the gene content of SGI1-C differs from those of the deletion mutants, no significant difference was observed in their transferability (Fig. 6). Previously, it was repeatedly observed that, in some transconjugants, more than one SGI1 copy integrated into the chromosome in direct orientation forming a concatemer^51^ and our unpublished data), therefore it was hypothesized that this phenomenon might be affected by an active RS.

**Fig. 7:**
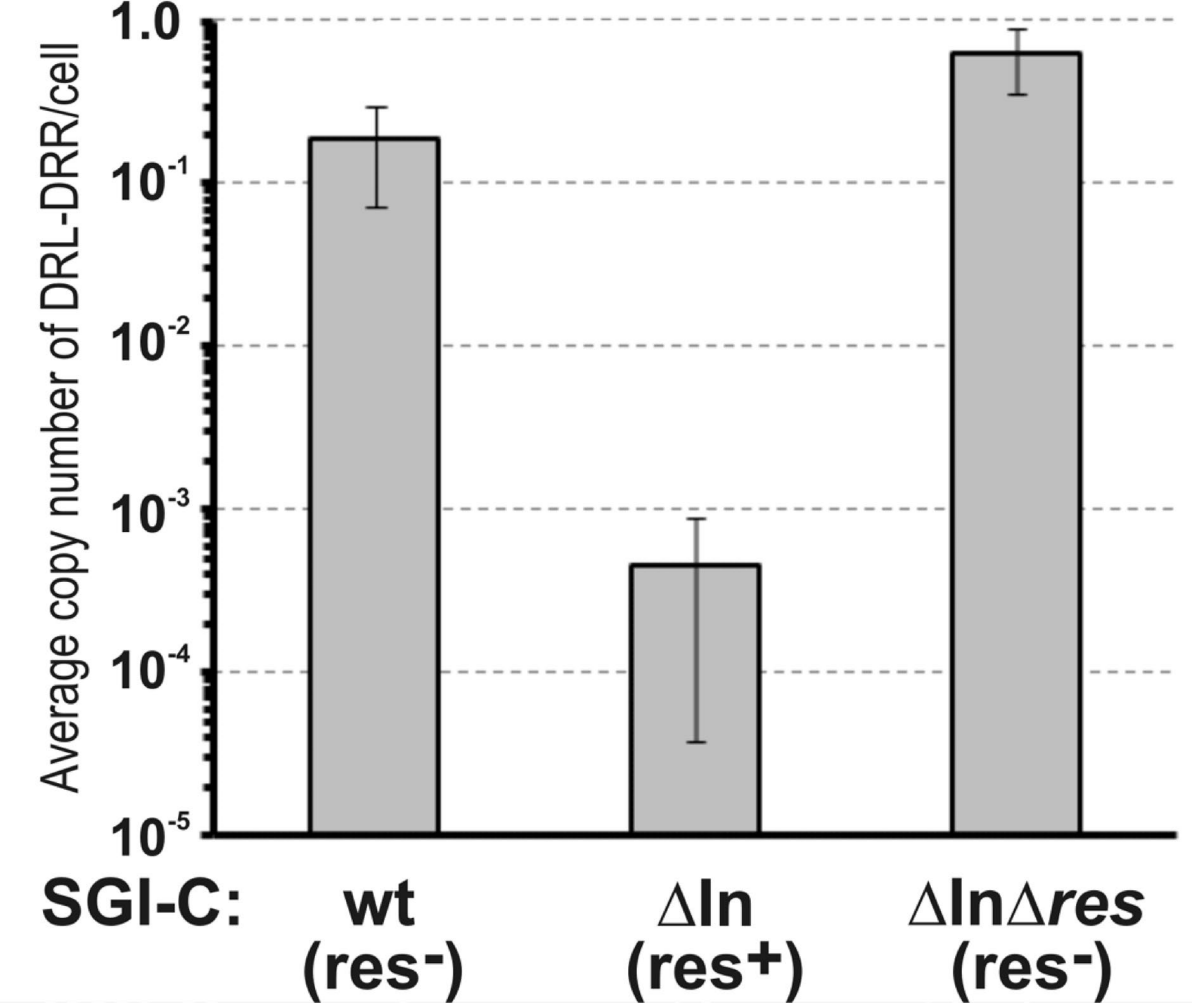
Average copy number of the DRL-DRR junctions of wt (res^−^), ΔIn (res^+^) and ΔInΔ*res* (res^−^) SGI1-C variants in the cell populations of randomly selected SGI1 multimer-containing clones. RT-qPCR was carried out for five DRL-DRR^+^ clones deriving from large-scale mating assays presented on (Fig. S5). Data of RT-qPCR were normalized to the single copy gene *trmE*. Note that all cells contain at least one SGI1 copy (monomer) and the measured average copy number of DRL-DRR junctions refers to the abundance of SGI1 multimers.

To quantify the frequency of concatemer formation, a 96-well plate-adapted mating method was applied that provided a large number of definitely independent SGI1-transconjugants. A single transconjugant colony from each individual mating was picked and pre-screened by a resistance test to detect the absence of the helper plasmid. This step was included because in the presence of a helper plasmid, SGI1 is excised and circularized forming an *attP* site equivalent to the DRL-DRR junction(s) (joined left and right direct repeats) occurring within concatemers. Then, R55-free colonies were cultured and tested by PCR for the regular SGI1 integration into *attB* site in *trmE* gene by amplification of *trmE*-DRL junction, and for the presence or absence of SGI1 concatemers by amplification of DRL-DRR junction using primers facing outward from SGI1 ends. This assay was repeated several times for wt, ΔIn and ΔInΔ*res* SGI1 and the results showed that the two resolution deficient SGI1 (wt and ΔInΔ*res*) produce significantly more concatemers (approx. 30% of the transconjugants) than SGI1ΔIn with the re-activated RS (approx. 14%) (Table 5, Fig. S5). Although it was not a quantitative PCR, beyond the lower number of res^+^ concatemers, the PCR amplicons were also more faint, indicating the smaller quantity of DRL-DRR junctions compared to that of res^−^ concatemer-bearing cultures (Fig. S5). Since the transconjugants were cultured under standardized conditions, i.e. they underwent approximately equal number of cell cycles, this observation suggested that the decay of res^+^ SGI1 concatemers is faster than those of res^−^. This conclusion was supported by passaging of concatemer-bearing transconjugants of SGI1ΔIn and SGI1ΔInΔ*res*. While DRL-DRR junctions were still detectable in SGI1ΔInΔ*res* (res^−^) transconjugants after the second passage (~ 14 generations), they disappeared from SGI1ΔIn (res^+^) transconjugants after the first passage (Fig. S6a,b). Similar results were obtained by real time qPCR, confirming that SGI1ΔInΔ*res* multimer-bearing cultures retained orders of magnitude larger amounts of concatemers than those containing SGI1ΔIn multimers (Fig. 7). It should be noted, however, that the primary multimer-containing transconjugant cells underwent up to 40–50 cell cycles before PCR detection due to the used methodology, during which concatemers may be resolved not only by the RS, but also by homologous recombination. Therefore, the progeny population of a multimer-carrying founder cell is a mixture of monomer- and multimer-containing cells, in which the proportion of multimers constantly decreases, even in the absence of an active RS. According to the qPCR estimation, the average copy number of DRL-DRR junctions was approx. 0.2–0.6 junctions/cell in the resolution deficient variants, but only approx. 0.0005 junctions/cell in the res^+^ SGI1ΔIn. In conclusion, the functional RS significantly accelerates the disappearance of SGI1 multimers.

**Table 5:** Frequency of SGI1-multimers in transconjugants.

SGI1-C	Transconjugants analysed ^*a*^		Frequency of multimers (%)
	*trmE*-DRL^+^	DRL-DRR^+^	
wt	96	22	22.9
	96	28	29.2
	93	44	47.3
	96	20	20.8
	96	25	26.0
	Mean and st. dev.		29.3 ± 9.5
ΔIn	93	15	16.1
	94	14	14.9
	96	15	15.6
	95	9	9.5
	Mean and st. dev., P value ^*b*^		14.0 ± 2.7, *P* = 0.028
ΔInΔ*res*	96	28	29.2
	96	34	35.4
	96	24	25.0
	Mean and st. dev., P value ^*b*^		29.9 ± 4.3, *P* = 0.931

^*a*^All analysed transconjugants were free of R55.

^*b*^P values of T-tests comparing the data of ΔIn and ΔInΔ*res* to that of wt.

## Discussion

Like in the majority of SGI1-REs, SGI1-C carries In104 upstream of the *res* gene. It is located 71 bp from the start codon of *res* and surrounded by ACTTG repeats, suggesting its transpositional acquisition and the disruption of the putative *res* site of the RS. In this study, In104, along with one copy of the bordering direct repeats, was precisely deleted from SGI1-C, and the functionality and the effects of the restored RS on SGI1 transfer were analysed. Gel shift experiments proved that the intergenic region between *res* and S044 genes (reconstituted after In104 deletion) comprises a *res* site that is bound by the Res protein expressed from the *res* gene of SGI1-C. Consistent with the overall structure of tripartite *res* sites, three complexes were detected (Fig. S2). For a more accurate identification of the *res* site and its subsites, the intergenic sequence was compared to several well-defined *res* sites of Tn*3*-family transposons. This alignment, which revealed that the closest known relatives of the SGI1-derived *res* site are found in Tn*3* and γδ transposons (Fig. 2), also suggested that the 28-bp boxI and the 30-bp boxII is separated by a 22-bp spacer, while spacing between boxII and the 25-bp boxIII is only 2-bp. The distance between the central positions of boxI and boxII is 51 bp, corresponding to 5 helical turns. Although this organization matches the general rule for Tn*3*-family *res* sites^39^, some differences can also be seen. The central bases in boxI defining the cleavage site is AC instead of AT present in boxI of Tn*3* and γδ *res* site. BoxII appears to be only 30 bp long instead of 34 bp and locates closer to boxIII, which, in turn, shows very weak symmetry (Fig. 2b,c). EMSA experiments confirmed that the predicted minimal full-length *res* site contains all three subsites (Fig. 3a). Using DNA-probes representing each individual subsite or their combination indicated that the Res protein binds the single boxI and boxIII, but recognition of boxII requires the presence of boxIII, suggesting a cooperative filling of these subsites by Res proteins (Fig. 3b–g). On the other hand, more than three shifted bands with the full *res* site, more than one complexes per subsites or binding a half subsite, which were observed in cases of Tn*3* and γδ resolvases and cognate *res* sites^52–54^ could not be detected under the applied conditions. Although these EMSAs aimed only to confirm the predictions for the restored *res* site and its subsites without detailed dissection of the recombination mechanisms, the results defined the binding sequences of SGI1 Res with a reasonable accuracy. This also implies that the original *res* site of SGI1 RS is interrupted by In104, which is integrated into the 22-bp spacer sequence between boxI and boxII, closer to boxII (Fig. 3h). Consistent with this conclusion, analyses of the target specificity of *res* site hunter transposons showed that their preferred targets are mostly located in the spacing between boxI and boxII subsites, but in several cases the subsites themselves were targets^31,32^ as observed with some In4-related integrons^26,37^.

In addition to sequence homology, the genetic constitution of SGI1 RS also corresponds to that of Tn*3*-type canonical systems regarding the location and orientation of the *res* site and the *res* gene^39^i.e. boxIII is located adjacent to the start codon of *res* gene, while boxI is distal to it. Using a promoter prediction tool^55^ and manual search, presumed − 10 and − 35 promoter boxes overlapping boxII were found (Fig. 2b). The location of this putative promoter is consistent with those identified for the *res* genes of Tn*3* and γδ^45,56^. The upstream location of In104 relative to this promoter suggests that it has no negative affect on the resolvase expression in SGI1. On the other hand, interruption of the *res* site presumably destroys its recombination ability since boxI is placed far away from boxII-III due to In104 insertion. The fact that no recombination was detected between two subsite I sequences in absence of boxII and boxIII in Tn*3*^57^, implies that separation of the subsites in RSs of SGI1-REs harbouring In104 in the *res* site^3^ inactivates these RSs, even in the presence of a functional Res protein. Interestingly, the *res* site of Uvp1 resolvase on plasmid R46 partially overlaps with the 5’-conserved segment of the In1 integron, i.e. boxI and boxII subsites lie within the 5’-CS^58 ^suggesting that integron insertion may exceptionally create an active *res* site, or prolonged evolution may generate a functional hybrid *res* site.

To prove the catalytic activity of SGI1-encoded resolvase, plasmid-based recombination systems were established. As expected, a very low recombination activity was observed when the *res* sites were placed on two compatible plasmids (Table 1). Even though several different cointegrates were obtained with and without Res expression (Fig. 4), correct structures deriving from site-specific recombination between the *res* sites of parental plasmids were found only in the presence of Res protein. Although the cointegrate formation is the reverse of the process for which the RSs have been evolved, it has also been observed for RSs of Tn*3*^40^ and pKLH2^41^. In contrast, the efficiency of cointegrate resolution was close to 100% (Tables 2, 3 and 4; Fig. 5b–d) indicating that SGI1 Res is a fully active resolvase. The in vitro resolution assay further supported this, however, the cointegrate decomposition was less efficient (Fig. 5f) possibly due to suboptimal conditions.

These results confirmed that the In104-deleted SGI1-C possesses an active RS, however it had no detectable impact on the conjugation, as transfer frequency of the res^+^ SGI1ΔIn did not differ significantly from those of the res^−^ wt SGI1-C and SGI1ΔInΔ*res* (Fig. 6), indicating that the resolution system has no considerable role in SGI1 mobilization. Interestingly, SGI1 tandem arrays carrying up to six copies at the primary *attB* site in *trmE* of *Salmonella* Typhimurium LT2 and a secondary site between *sodB* and *purE* have been reported^51^and were also observed occassionally in our previous mating experiments. Such tandemly repeated SGI1 copies can serve as substrates for the RS, so a large-scale mating assay providing undoubtedly independent transconjugants was applied to estimate the incidence of concatemers. Analysis of several hundreds of such transconjugants revealed a striking difference in the frequency of multimers in transconjugant colonies having res^−^ or res^+^ SGI1: approx. 30% of wt SGI1-C and SGI1ΔInΔ*res* transconjugants carried more than one SGI1 copies, while this rate was around 14% for SGI1ΔIn (Table 5), where intensity of PCR signals indicative of DRL-DRR junctions was also much lower than in cases of res^−^ variants (Fig. S5). Considering that the descendants of the original transconjugant cells have undergone about 50 cell divisions before PCR detection, it can be concluded that during this time the reactivated RS completely or, in some clones, almost completely eliminated the concatemers. Passaging of concatemer-bearing populations proved that DRL-DRR junctions of SGI1ΔIn became undetectable after an additional passage (~ 7 generations), while those of res^−^ variants gave robust PCR signals even after two additional passages (Fig. S5). Quantitative PCR for several multimer-containing clones also confirmed that the average number of junctions per cell is approx. 3 orders of magnitude larger in the res^−^ wt SGI1-C and SGI1ΔInΔ*res* than in the res^+^ SGI1ΔIn concatemer-bearing clones after equal number of cell cycles (0.2–0.6 vs. ~0.0005/cell, Fig. 7). This means that the average copy number in SGI1 arrays in a cell population derived from a multimer-containing founder cell was 1.2–1.6 for the res^−^, but very close to 1 for the res^+^ SGI1 variant, indicating that the active RS highly accelerates the conversion of SGI1 multimers into monomers. It is noteworthy that homologous recombination can also reduce the copy number of multimers during propagation as was also suggested^51^, therefore, the progeny population of a multimer-carrying founder cell is always a mixture of monomer- and multimer-containing cells, where the proportion of concatemers is continuously decreasing regardless of the activity of the RS, although our results show that the resolution by homologous recombination is orders of magnitude slower. In any case, if there is no selective advantage of multimer-containing clones, normal growth conditions likely favour cells without extra SGI1 copies, thus, the res^−^ SGI1 multimers will also disappear from the population after a while, but their lif-time is much longer.

An interesting question is how SGI1 concatemers arise and whether they play a role in SGI1 spread and evolution. At least three processes can be responsible for concatemer formation. (i) It is well known that homologous recombination can create plasmid dimers (or even multimers) from multicopy plasmids causing significant stability concerns, against which plasmids defend themselves by multiple stabilizing mechanisms such as dimer resolution, partitioning and addiction systems^38,59,60^. Since SGI1 exists as an extrachromosomal circular form in 7–8 copies in the presence of IncC plasmids^9,18^, multimers can be formed by homologous recombination in donor cells, and then transferred and integrated in the recipient cell just like the monomer form. (ii) Theoretically, relaxase-dependent rolling circle replication in donor cells occurring during conjugal transfer may also generate multimers if replication is not correctly terminated at the *oriT* due to mutations or other factors^61,62^. Although molecular details of SGI1 transfer initiation and termination are not yet known, the Tyr-recombinase-family protein, MpsA, presumably acts as an atypical relaxase. Furthermore, the relaxase of IncC helpers proved to facilitate SGI1 transfer^12^. As a consequence, imperfect cooperation between IncC and SGI1 transfer components or incorrect termination by the atypical relaxase might lead to formation and transfer of SGI1 multimers. (iii) Finally, consecutive integration events of SGI1 monomers next to one another can also result in concatemers in the recipient cell. Integration of SGI1 into *attB* results in the formation of direct repeats, DRL and DRR, flanking the element. The 18-bp DRL sequence is identical to the joined ends of the free circular SGI1, named *attP*, while the former *attB* is preserved in DRR^63^. Therefore, DRR can serve as a target site (*attB*) for a second or multiple integrations. Mixed arrays of SGI1 and different GIs have been reported^3^, which can only be explained by consecutive integration events, however this possibility has not yet been proven for SGI1 multimers.

The fact that wt SGI1-C behaved very similarly to the definitely resolution deficient SGI1ΔInΔ*res* in all experiments suggests that RS is inactive in SGI1-C and consequently in all SGI1-REs where In104 is integrated at the same or adjacent positions of the *res* site. Although a number of In104-free SGI1-REs, mostly found in water-born host organisms, presumably possess an active RS, those with inactive RS are in striking majority^3^. Their increased prevalence can be explained by the selective advantage of the MDR region under the pressure of intensive use of antibiotics in the last decades. However, one would expect that antibiotic selection favours MDR SGI1-REs equally, regardless of the position of the MDR cluster. In contrast, the resolution deficient MDR SGI1-REs appear much more common, as only one example, SGI2, is known, whose resolution system is almost certainly functional and also has an MDR region. SGI2 *res* gene is identical to that of SGI1 and only two base substitutions are present in the *res* site: an A→T change in boxII and a G→A change in the 2-bp spacer between boxII and boxIII, which do not affect the promoter boxes of *res* gene and presumably the Res-binding to boxII. Interestingly, the closest relatives of SGI2, found in *S. enterica* Enteritidis 92–0392 and Hadar FNE0129, also contain the In104 cluster at their *res* site^3^. Why SGI2 could not become so frequent? Alternative explanations could be that SGI2 is very recent and has not had enough time to spread, or that inactivation of the helicase gene (S023) has adversely affected its spread or stability.

Our opinion is that inactivation of the RS may be beneficial for SGI1-REs in evolutionary timescale, leading to higher frequency of resolution deficient variants. Increased persistence of multimers may have some biological cost, on the other hand, it can exert higher level of antibiotic resistance for the recipient cell due to the dosage effect^64 ^which is advantageous especially under selection pressure caused by the use of antibiotics. Furthermore, a higher copy number of SGI1 strengthens its incompatibility with the helper plasmids^14^ which are destabilizing factors for SGI1^11^. Therefore, multimers may enhance the stability of SGI1 in the recipient cells by more efficiently expelling the helper plasmid.

The facts that different positions of In104 were found among MDR SGI1-REs (three in SGI1 cluster, five in AGI1 cluster and one more in PGI1 cluster)^3^ and the occurrence of SGI2 variants having In104 in different positions (S023, *res* site), strongly suggest that inactivation of RS by In104 insertion occurred independently in different lineages. Furthermore, several elements lack the RS due to deletions possibly occurred after In104 integration^3^. Although it cannot be excluded that the selective advantage of MDR variants was the antibiotic resistance conferred by the acquired integron and not the inactivation of RS itself, the rarity of SGI2-like elements, containing both In104 and active RS, seems to contradict this. Although the current ratio of active/inactive RSs in known SGI1-REs may have been significantly influenced by genetic drift and past short-term selection events, we believe that more frequent formation and longer persistence of multimers, particularly over evolutionary timescales, may also have played an important role in the increased abundance of SGI1-REs containing inactive RSs.

The function of RSs is obvious in the case of Tn*3*-like transposons that translocate via a replicative way generating cointegrates, however, the role of related systems in the chromosomally integrated SGI1-like elements is unclear. The prevalence of RSs in the SGI1 family and the occurrence of two different forms suggest that their acquisition might have conferred a selection advantage. However, this raises a further question: what was the advantage of the initially active RS for the ancient SGI1 if it is now mostly inactive, and this does not appear to be detrimental to the spread of such elements, in fact, they constitute the vast majority. Further in-depth analysis of the SGI1-REs is needed to answer these questions and better understand the evolution of these elements.

## Methods

### DNA and microbial techniques

Standard molecular biology procedures were carried out according to^65^. Chemicals were purchased from Merck, Roth and Reanal. DNA fragments for cloning or gene replacement recombination were amplified using Pwo (Roche) and Phusion (Thermo Fisher Scientific) high-fidelity DNA polymerases. Enzymes for cloning and PCRs were purchased from Thermo Fisher Scientific, New England Biolabs and Merck. For cloning purposes, *E. coli* strain TG1 was used. Cloned PCR products, as well as junctions in plasmid cointegrates were sequenced on an ABI 3500xL Genetic Analyzer (Life Technologies). Plasmid features are listed in Table S1, while detailed methodology for plasmid construction is described in Text S1.

Oligonucleotides used in this work are listed in Table S2. Primers annealing to SGI1 were designed using the published sequence of SGI1 (GenBank: AF261825). Test/colony PCRs were performed as described previously^66^. For colony PCRs, single colonies were picked with a pipette tip and suspended in 100 µl 0.9% NaCl solution or in 100 µl LB supplemented with appropriate antibiotics in 96-well plates, grown 4 h at 37 °C, and then 2.5 µl bacterial suspension was used as template in PCRs.

*E. coli* strains (listed in Table S3) were routinely grown at 37 °C in Luria Bertani (LB) broth/agar supplemented with the appropriate antibiotics at the following final concentrations: ampicillin (Ap) 150 µg/ml, chloramphenicol (Cm) 20 µg/ml, kanamycin (Km) 30 µg/ml, nalidixic acid (Nal) 20 µg/ml, spectinomycin (Sp) 50 µg/ml, streptomycin (Sm) 50 µg/ml, and tetracycline (Tc) 10 µg/ml.

### Construction of SGI1ΔIn and SGI1ΔInΔ*res*

SGI1ΔIn was constructed in three consecutive steps. First, the entire In104 cluster with one copy of its flanking 5-bp DRs was removed. The KO PCR fragment for the scarless deletion of In104 was amplified from pSG76-CS^42^ template plasmid using primers delIn104AB and delIn104C. Gene replacement was induced by λ Red recombinase expressed from pKD46 in *E. coli* strain TG1Nal::SGI1-C^11,13^ resulting in a Cm^R^Sm/Sp^S^ SGI1ΔIn variant (SGI1ΔIn::Cm^R^). Then, a Sm/Sp^R^ marker gene (*aadA1*) was inserted downstream of S023. The resistance cassette was amplified from pGMY1 template plasmid using Sm/Spinsfor and Sm/Spinsrev primers and the amplicon was integrated into SGI1ΔIn::Cm^R^ via λ Red induced recombination leading to SGI1::Sm/Sp^R^ΔIn::Cm^R^. Finally, after removal of the thermosensitive pKD46 at 42 °C, the Cm^R^ gene was eliminated by DSB-stimulated recombinational repair process facilitated by I-*Sce*I cleavage^42^. Expression of I-*Sce*I from pMSZ934 was induced by 30 µg/ml chlortetracycline ON at 30 °C in LB + Sm + Km + Ap broth. The Cm^S^ clones were selected by replica plating and the scarless deletion in the resulting Sm/Sp^R^Cm^S^ SGI1ΔIn variant was verified by amplifying and sequencing the region surrounding the deletion site using primers delIn104seqfor and d043seqrev.

The *res* gene (S027) was deleted from SGI1ΔIn by the one-step recombination method^67^. The Cm^R^ KO PCR fragment was amplified from pKD3 template plasmid^67^ with 027delfor and 027delrev primers. Integration was induced by the expression of λ Red recombinase from pKD46, then the Cm^R^ cassette was removed by the FLP recombinase produced from pCP20.

PCR amplicons for chromosomal integration were electroporated using a BTX Electro Cell Manipulator 600 and 2-mm gap electroporation cuvettes as described previously^68^. Incubation at 30 °C and 42 °C was applied to maintain and cure plasmids with a temperature-sensitive pSC101 replication system (pKD46, pMSZ934, pCP20).

### Purification of the resolvase protein

*E. coli* strain BL21 (DE3) transformed with a pET16b-based Res-producer plasmid pAVE22 was grown overnight (ON) in 20 ml LB + Ap + 0.1% glucose. 240 ml fresh LB + Ap broth was inoculated with 16 ml of ON culture and incubated at 37ºC for 30 min, then induced with 0.2 mM IPTG and grown for an additional 3 h under vigorous shaking. Bacteria were harvested by centrifugation and the Res protein was purified under native conditions according to the protocol of QIAexpress Ni-NTA Fast start 6×His-tagged Kit (Qiagen). Samples were taken during the procedure and analysed by denaturing polyacrylamide gel electrophoresis in the presence of Spectra Multicolour Broad Range protein ladder (Thermo Fisher Scientific) using NuPAGE 4–12% gradient polyacrylamide gels according to the manufacturer’s recommendations (Thermo Fisher Scientific). Gels were stained with InstantBlue (Expedeon). Purified Res protein (approx. 0.3 µg/µl) was stored at -80 ºC in 50 µl aliquots until use. Protein concentration was measured using the Qubit Protein Assay Kit and Qubit 3.0 instrument (Life Technologies). The same method was applied for purification of a negative control protein sample from BL21 (DE3)/pET16b strain expressing no Res protein.

### Electrophoretic mobility shift assays (EMSA)

Fluorescent DNA probes for EMSAs were amplified with primers labelled with 6-carboxyfluorescein (6-FAM) at 5′ end. The high copy plasmid pEMBL19 and its derivatives, containing the entire *res* site or parts of it (pAVE18, pAVE24 – pAVE33, Table S1), served as templates. For amplification of the entire S027-S044 intergenic region (207 bp) and the predicted *res* site (122 bp), pAVE18 template plasmid and primer pairs EMSA_res_F_5’FAM – EMSA_res_R_5’FAM and EMSA_for2_5’FAM – EMSA_rev2_5’FAM were used, respectively. Partial *res* site probes (half boxI, boxI, II, III and their combinations) were amplified using primers pucfor24_5′FAM and pucrev25_5′FAM annealing to the flanking regions of multicloning site of pEMBL19, the basic vector for template plasmids pAVE24 – pAVE33 (probes are listed in Table S4). Labelled amplicons were purified on 6% non-denaturing polyacrylamide gel and eluted from gel slices by the crush and soak method^69^. Briefly, the labelled probes were excised from the gel after 2 h run at 8 V/cm, gel slices were crushed and ON incubated at 37 ºC in 0.6 ml isolation buffer (10 mM Tris HCl, pH 8; 1 mM EDTA; 0.2% SDS; 0.3 M NaCl) under shaking, then DNA was ethanol-precipitated from the supernatant and dissolved in 20 µl EB buffer (QIAgen).

Binding reactions were performed in a final volume of 20 µl containing 25 mM Tris HCl pH 8.0, 50 mM NaCl, 50 mM KCl, 1 mM EDTA, 1 mM DTT), 5% glycerol, and 5.6 ng of full *res* site probes, 8 ng of partial *res* site probes and 4 ng of half boxI probe. In addition, increasing amounts of purified Res protein were added to each mixture followed by a 20 min incubation at 37 ºC. To test the binding specificity, approx. 1.7 µg (~ 300-fold) of unlabelled DNA, and 10 min later the labelled probe was added to the binding reaction mixture. As a negative control, protein sample purified from BL21 (DE3)/pET16b strain (no Res) was also used. After pre-running for 45 min at room temperature (RT), samples were loaded onto a 6% non-denaturing polyacrylamide gel and run for 3 h at 8 V/cm at 4 ºC in 1× TBE buffer. The fluorescence signals were detected using a ChemiDoc^™^ MP Imaging System (Bio-Rad).

### Detection of cointegrate formation in vivo

To investigate whether the Res protein catalyses the recombination, a three-plasmid system was developed. At first, *recA* strains TG2 λ*pir* and S17-1 λ*pir* were co-transformed with the compatible, full *res* site-containing parental plasmids pJKI1088 (p15A *rep ori*, Km^R^, P1) and pJKI1092 (R6Kγ *rep ori*, Cm^R^, P2). Then, four Km^R^Cm^R^ transformant colonies of both strains were selected to introduce the Res-producing pAVE20 or the non-producing empty vector pKK223-3 (negative control) by a second transformation. Transformants containing the three plasmids were selected on LB + Km + Cm + Ap plates incubated ON at 37 °C. Expression of the Res protein from pAVE20 was ensured by leaking of P_tac_ promoter without IPTG induction. Plasmid DNA was isolated from four parallel Km^R^Cm^R^Ap^R^ colonies of each transformation and introduced into TG1 strain where replication of the R6Kγ-based Cm^R^ pJKI1092 was inhibited (i.e. Cm^R^ colonies can be observed only if pJKI1092 is fused by recombination to the p15A-based Km^R^ plasmid pJKI1088 that ensures replication of the cointegrate). Four individually transformed cultures were grown 1 h at 37 °C in LB broth, then, titered on LB + Km and LB + Km + Cm plates incubated ON at 37 °C and frequency of cointegrates were calculated as titers of Km^R^Cm^R^/Km^R^ colonies. Km^R^Cm^R^ colonies then were pre-screened by colony PCRs using primer pairs FRTfor-pBRBgl and pucrev25-cat3.2 amplifying the expected junctions formed by recombination between *res* sites of the parental plasmids. Plasmid DNA was isolated from all Km^R^Cm^R^ colonies giving positive signal for at least one of the two junctions and, in cases of the Res^−^ controls, some randomly selected Km^R^Cm^R^ clones were also examined. The cointegrate structure was analysed by restriction mapping and the correct junctions were confirmed by sequencing.

### Cointegrate resolution in vivo

*E. coli* strains TG1 and S17-1 λ*pir*, both containing pJKI1099 cointegrate were transformed with the Res-producer, pAVE20 or the empty vector pKK223-3 (negative control). After 1 h growth of four parallel transformations at 37 °C in LB broth, transformants were titrated on LB, LB + Km + Ap, and LB + Km + Cm + Ap plates. TG1 colonies grown on LB + Km + Ap plates were replica-plated onto LB + Km + Ap and LB + Km + Cm + Ap to determine the ratio of cointegrates that remained intact in the presence or absence of the Res-producer or control plasmids. Plasmid content of selected colonies was analysed by restriction mapping and PCRs amplifying the junctions of the cointegrate and the restored *res* sites of the parental plasmids (see also the text).

Plasmid DNA of the analysed Res^+^ and Res^−^ Km^R^Cm^R^Ap^R^ S17-1 λ*pir* colonies were introduced into S17-1 λ*pir* host, transformants were spread onto LB + Km and LB + Cm plates and the colonies obtained after ON incubation at 37 °C were replica-plated onto LB + Km + Cm plates to determine the frequency of intact cointegrates in the original plasmid populations.

### Cointegrate resolution assay in vitro

Plasmid DNA for in vitro assay was purified using the QIAgen Plasmid Miniprep kit according to the manufacturer’s protocol. Approx. 200 ng of cointegrate DNA (pJKI1099) was incubated with or without ~ 2.7 µg purified His-tagged Res protein for 20 min at 37 °C in a final volume of 20 µl reaction mixture (25 mM Tris HCl pH 8.0, 50 mM NaCl, 50 mM KCl, 1 mM EDTA and 1 mM DTT). DNA was then phenol-chloroform-extracted, dissolved in 6 µl of TE buffer (10 mM Tris, 1 mM EDTA, pH 8.0), and digested with 10 units of *Sca*I. Samples and *Sca*I-digested control plasmids (P1 and P2) were run on 1% agarose gel in 1×TBE buffer. Gel was stained with SybrGreen (Sigma) diluted 1:10000 in TBE, and scanned using a ChemiDoc^™^ MP Imaging System. Relative quantification of the fragments were carried out Relative Quantity Tools of Image Lab 5.2.1 (BioRad) software package.

### Mating assays

Mating experiments to determine the transfer frequencies of SGI1 variants were carried out with some modifications as described previously^13^. *E. coli* strain TG90 (Tc^R^) was used as recipient with TG1Nal-derived donor strains containing SGI1-C (wt), SGI1ΔIn, or SGI1ΔInΔ*res* and the IncC Type 2 plasmid R55, which was freshly conjugated from TG2/R55 strain^70^. 300 µl ON cultures of donor strains grown in LB + Nal + Sm + Sp + Cm (approx. 1 × 10^9^ cells/ml) and 100 µl ON culture of the recipient strain grown in LB + Tc (approx. 2–3 × 10^9^ cells/ml) were mixed, centrifuged for 1 min at 10,000 rpm at RT, resuspended in 0.5 ml 0.9% NaCl solution. Then, 20 µl was spread on 1.5 ml LB agar poured into the wells of a 24-well plate and incubated for 2 h at 37 °C. The bacterial lawn was resuspended in 150 µl 0.9% NaCl solution, serially diluted 1:10 and titrated on LB + Nal + Sp + Cm (donor), LB + Tc (recipient) and LB + Tc + Sp (transconjugant) agar plates. Transfer frequencies were calculated as the ratio of transconjugant per donor or recipient titers.

A modified, 96-well plate-adapted mating method was used to gain definitely independent transconjugants in large-scale to examine the frequency of SGI1 multimers. TG1Nal donor strains containing one of the SGI1 variants along with R55, and TG90 recipient strain were cultured ON in 4 ml LB broth supplemented with appropriate antibiotics. 3 ml of donor and 1 ml of recipient cultures were spin down (1 min, 10,000 rpm, RT), and the cells were resuspended in 10 ml LB, then, 100 µl of donor and recipient suspension was dispensed with a multichannel pipette into a 96-well plate and mixed by pipetting. After 1 h incubation at 37°C, 5 µl of the individual mating mixtures was transferred into another 96-well plate containing 200 µl of 0.9% NaCl solution and mixed by pipetting (40× dilution). Finally, 5 µl of the resulting suspensions from each well was dropped onto LB + Tc + Sp agar plates to select SGI1 transconjugants. After ON incubation at 37°C, a single colony from each drop was picked and tested for the lack of R55 (Cm^S^). This method provided 96 definitely independent transconjugants from each mating plate. Tc^R^Sm/Sp^R^Cm^S^ colonies were grown ON at 37°C in 150 µl LB + Tc + Sp broth in 96-well plates, then 2.5 µl of these cultures were used as template for colony PCRs. The *trmE*-DRL junction-specific primer pair attsgi1for – LJ2 were used to confirm the chromosomal SGI1 integration into the *attB* site at the 3’ end of the *trmE* gene, while LJ2 and RJ2 primers (both directed outward of SGI1 ends) were used to detect DRL-DRR junctions in clones harbouring head-to-tail SGI1 multimers.

To examine the stability of SGI1 multimers, 2 µl of DRL-DRR^+^ ON cultures (passage 0) was transferred into 200 µl LB + Tc + Sp broth dispensed into 96-well plates and incubated ON at 37 °C (approx. 6–7 generations), then the DRL-DRR-specific PCR was carried out again. Passaging was repeated twice by keeping the (still) positive clones.

### Real-time quantitative PCR (qPCR) for estimating the average copy number of wt, ΔIn and ΔInΔ*res* SGI1-C variants in SGI1 multimer containing clones

The average copy numbers of DRL-DRR junctions of wt, ΔIn and ΔInΔ*res* variants of SGI1 per cell in the SGI1-multimer-containing transconjugant populations were estimated by qPCR as described previously for quantification of the free circular SGI1 copies by detecting *attP*^9^, which is equivalent to the DRL-DRR junction. As circular SGI1 is formed in the presence of the helper plasmid, to avoid SGI1 excision and guarantee that the detected DRL-DRR junctions are from chromosomally integrated multimers, pre-screening of transconjugants by a resistance test was carried out to exclude R55-containing colonies. Thus, the ratio of DRL-DRR junctions and the single-copy chromosomal gene, *trmE*, was used to measure the average copy number of SGI1 concatemers in R55-free cell populations. Amounts of DRL-DRR junctions were normalized to the amount *trmE* in each sample and an *E. coli* strain (TG1Nal::*attP*_SGI1_) containing a single chromosomal copy of *attP*, which was used to calibrate the qPCR assay. The LJ3 – RJ5, and attsgifor2 – attsgirev3 primer pairs (Invitrogen, desalted) were used to amplify the 251- and 207-bp fragments specific for DRL-DRR junction and *trmE*, respectively. Specificity of amplicons was confirmed by generating melting curves.

Total DNAs for the qPCR were isolated from 2 ml ON cultures of DRL-DRR^+^ R55-free (Cm^S^) clones using the ExctractMe DNA & RNA Extraction kit (Blirt S. A.) according to the manufacturer’s protocol. DNA samples were stored at -20 °C and used after thawing on ice for qPCRs that were carried out in five biological and two technical replicates using 20 ng DNA as template. DNA concentrations were measured using NanoDrop 1000 Spectrophotometer. Reactions were performed in a final volume of 10 µl using a LightCycler^®^96 detection system (Roche). Reaction mixtures contained 1×qPCRBIO SyGreen Lo-ROX master mix (PCR Biosystems), 400 nM forward and reverse primers, and 20 ng total DNA as template. Cycling was as follows: initial denaturation at 95 °C for 2 min; 40 cycles of 95 °C for 5 s and 60 °C for 30 s with a ramp time of 2.2 °C/s. C_q_ was determined using Roche software (version 1.1.1.1320). Relative amounts of amplified DRL-DRR junctions were calculated based on the C_q_ deviation of experimental samples compared to the control sample (TG1Nal::*attP*_SGI1_) and were expressed relative to the calibrator single copy/cell (*trmE*) sequence. C_q_ of non-template controls were high (30.52 and 28.48 for DRL-DRR and *trmE*, respectively) compared to the control and experimental samples (ranged between 11.72 and 25.26 for DRL-DRR and 12.35–13.73 for *trmE*).

### Image processing, sequence alignment, statistical data evaluation

All figures were created using CorelDraw 10.0 software. Original gel images from the ChemiDoc™ MP Imaging System (Bio-Rad) were processed (conversion into black and white, changing brightness and contrast, cropping, removing dye spots from EMSAs with 5’FAM labelled probes) using Adobe Photoshop 6.0 without altering any relevant information of the images.

Sequence alignments were made using MultaAlin software (http://multalin.toulouse.inra.fr/multalin/)^71^. The phylogenetic reconstructions were made using MEGA7^72^. All homology searches were carried out using BLAST^73^ in the NCBI database (http://www.ncbi.nlm.nih.gov/). Graphs, as well as paired t-tests for significance analyses were generated using MS Excel 2007.

## Electronic supplementary material

Below is the link to the electronic supplementary material.

Supplementary Material 1

## Data Availability

All data is contained within the manuscript and/or the supplementary materials.

## Abbreviations

aa: Amino acid
AGI1: *Acinetobacter* Genomic Island 1
AR: Antibiotic resistance
DR: Direct repeat
DRL: 18-bp direct repeat bordering the left end of SGI1
DRR: 18-bp direct repeat bordering the right end of SGI1
EMSA: Electrophoretic mobility shift assay
In104: In4-type integron region of SGI1
IS: Insertion sequence
IR: Inverted repeat
LB: Luria-Bertani culture medium
MDR: Multidrug resistance
ORF: Open reading frame
*oriT*: Origin of conjugative transfer
*oriV*: Origin of replication
PGI1: *Proteus* Genomic Island 1
res^+^/res^−^: Resolution competent/deficient
*res*: Resolvase gene
*res * site: Recombination site of resolution systems
Res: Resolvase protein
Res^+^/Res^−^: Resolvase protein producer/non-producer
RS: Resolution system
SGI1: *Salmonella* genomic island 1
SGI1-C: C-variant of SGI1
SGI1ΔIn: SGI1 deleted for In104 integron
SGI1ΔInΔ*res*: SGI1 deleted for In104 integron and *res* gene
SGI1-RE: SGI1 related elements
TA: Toxin-antitoxin
Tn: Transposon
T4SS: Type IV secretion system
VGI: *Vibrio* genomic island
