# Supplementary material for: Restoration and functional analysis of the SGI1 resolution system – SGI1 multimers are eliminated by the reactivated resolution
Source: Sci Rep. 2025 Jul 1;15:20550. doi: 10.1038/s41598-025-06025-6 (PMC12217951; doi:10.1038/s41598-025-06025-6)
Supplement: Supplementary file 1 — Supplementary Material 1 [file 41598_2025_6025_MOESM1_ESM.pdf]

Additional Supplementary data and Methods

Content:

Supplementary Figure S1
Supplementary Figure S2
Supplementary Figure S3
Supplementary Figure S4
Supplementary Figure S5
Supplementary Figure S6
Supplementary Figure S7
Supplementary Figure S8
Supplementary Figure S9

Supplementary Table S1
Supplementary Table S2
Supplementary Table S3
Supplementary Table S4

Supplementary Text S1
Supplementary References

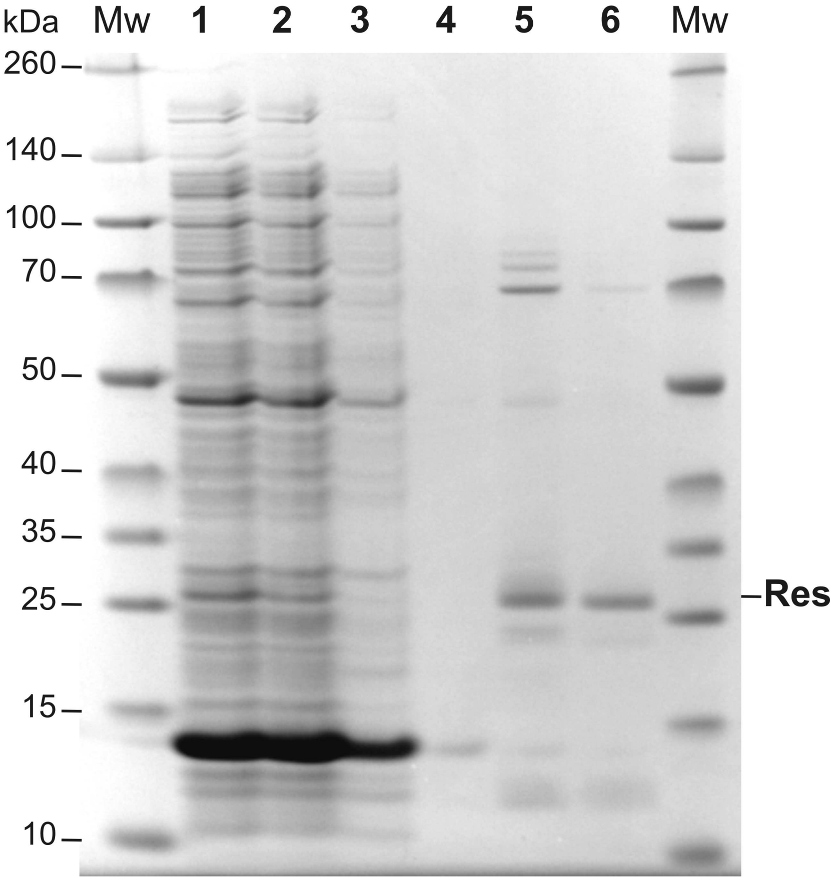

Suppl. Figure S1. Purification of His-tagged Res protein on Ni-resin. Res protein was purified from 0.2 mM IPTG-induced BL21 (DE) culture carrying the Res-producer plasmid, pAVE22. Lane 1 cleared lysate, lane 2 flow-through fraction, lanes 3 and 4 flow through fractions of washing step 1 and 2, lanes 5 and 6 eluted fractions, Mw - molecular-weight ladder (Spectra Multicolour Broad Range protein ladder). The 2nd elution fraction shown in lane 6 was used in EMSAs.

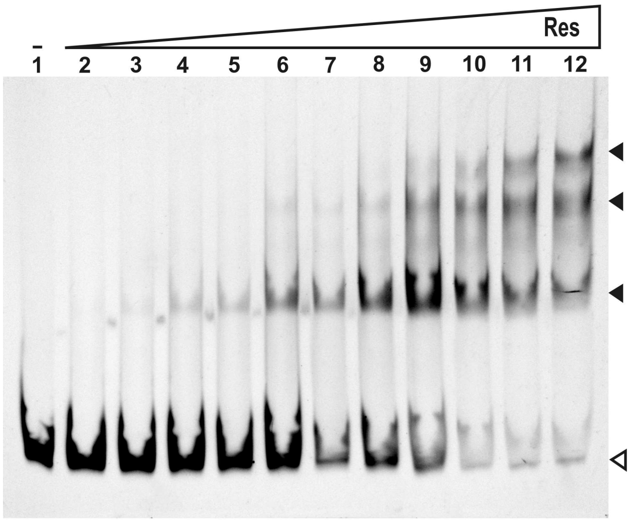

Suppl. Figure S2. EMSA for the detection of DNA-protein complexes formed upon binding the His-tagged resolvase (Res) to the restored *res* site. The 5'FAM-labelled 207-bp probe represented the intergenic region between the *res* and S044 genes including the restored *res* site of SGI1ΔIn. Lane 1 no Res, lanes 2-12 increasing amount of 10×His-Res protein: 0, 0.09, 0.18, 0.26, 0.35, 052, 0.70, 0.88, 1.23, 1.58, 2.10, and 3.15 μg. Open arrowhead points to the unbound probe, filled arrowheads indicate the DNA-protein complexes.

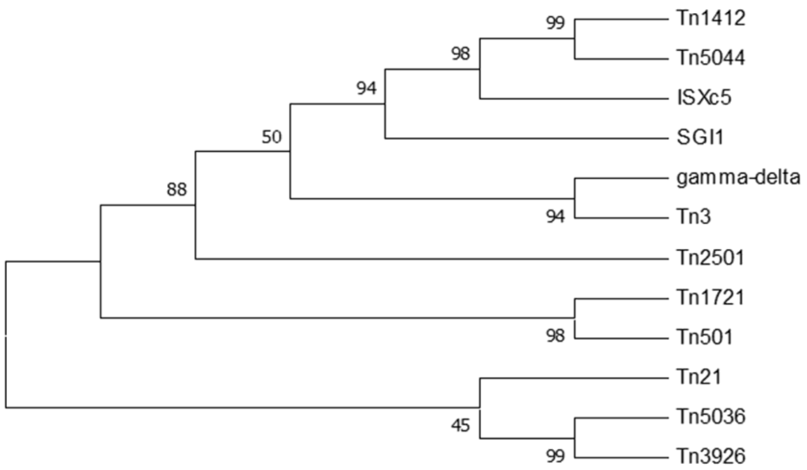

Suppl. Figure S3. Phylogenetic relationship of SGI1-encoded Res and eleven Tn-derived resolvases. The phylogenetic tree was generated by the Maximum likelihood method , bootstrapping was repeated 500 times.

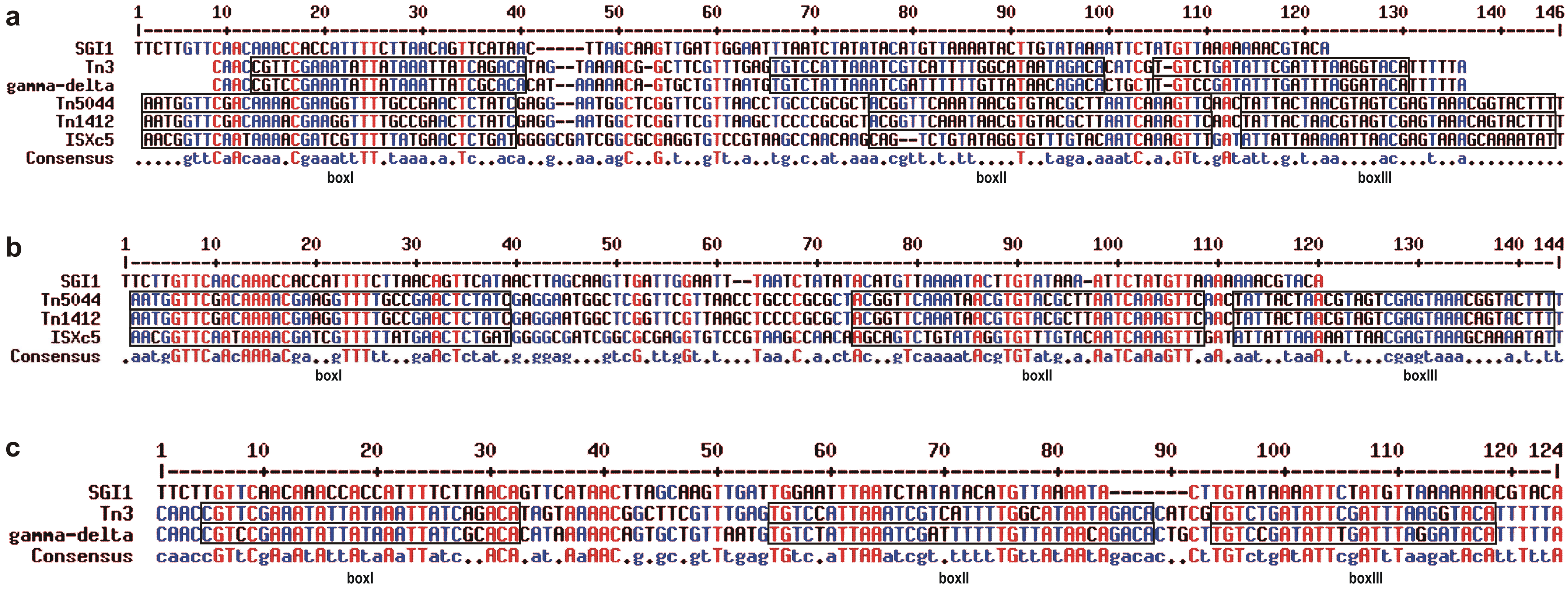

Suppl. Figure S4. Alignments of SGI1 *res* site with the five closest relative *res* sites. (a) Alignment with all five sites. (b) Alignment with sites of the Tn*5044* cluster. (c) Alignment with sites of the Tn*3* cluster. Resolvase binding sites in Tn*3*, γδ, Tn*5044*, Tn*1412* and IS*Xc3* *res* sites are boxed.

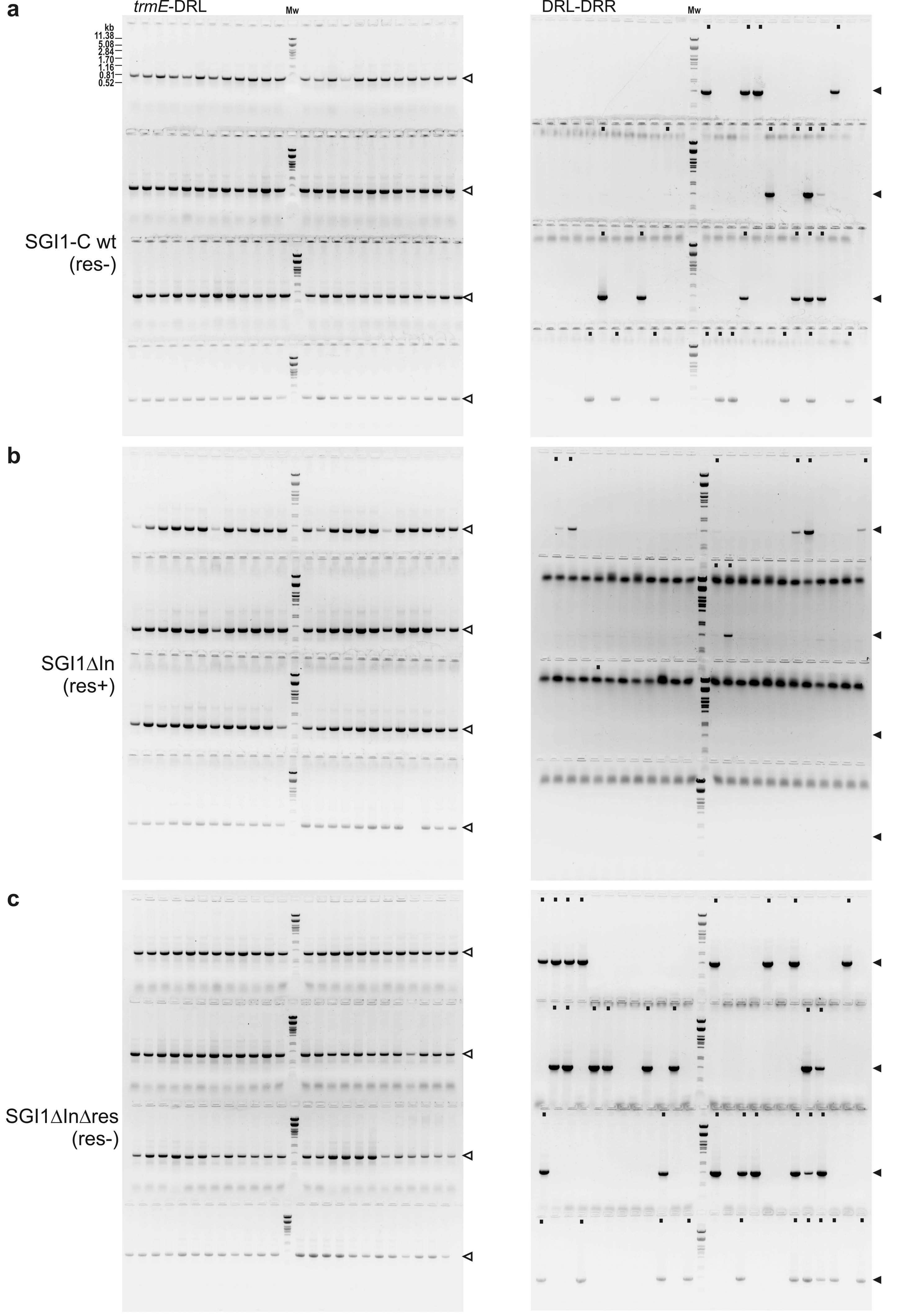

Suppl. Figure S5. Representative set of colony PCR analyses for the occurrance of multimeric SGI1 integration in independent R55 free transconjugants. (a) Wt SGI1-C transconjugants (res⁻, i.e. RS is inactive). (b) SGI1ΔIn transconjugants (res⁺, i. e. the RS is active). (c) SGI1ΔIn*Δres* transconjugants (res⁻). Left panels show the SGI1 integration into the *attB* site at the 3' end of *trmE* (open arrowheads point to the 699-bp amplicons of *trmE*-DRL juncion amplified with primers attsgi1for and LJ2). Right panels show the DRL-DRR junction indicating multimeric SGI1 integration (filled arrowheads point to the 800-bp amplicons of DRL-DRR junction amplified with primers LJ2 and RJ2). DRL-DRR+ clones are marked with dots. Mw molecular-weight marker (λDNA digested with *Pst*I).

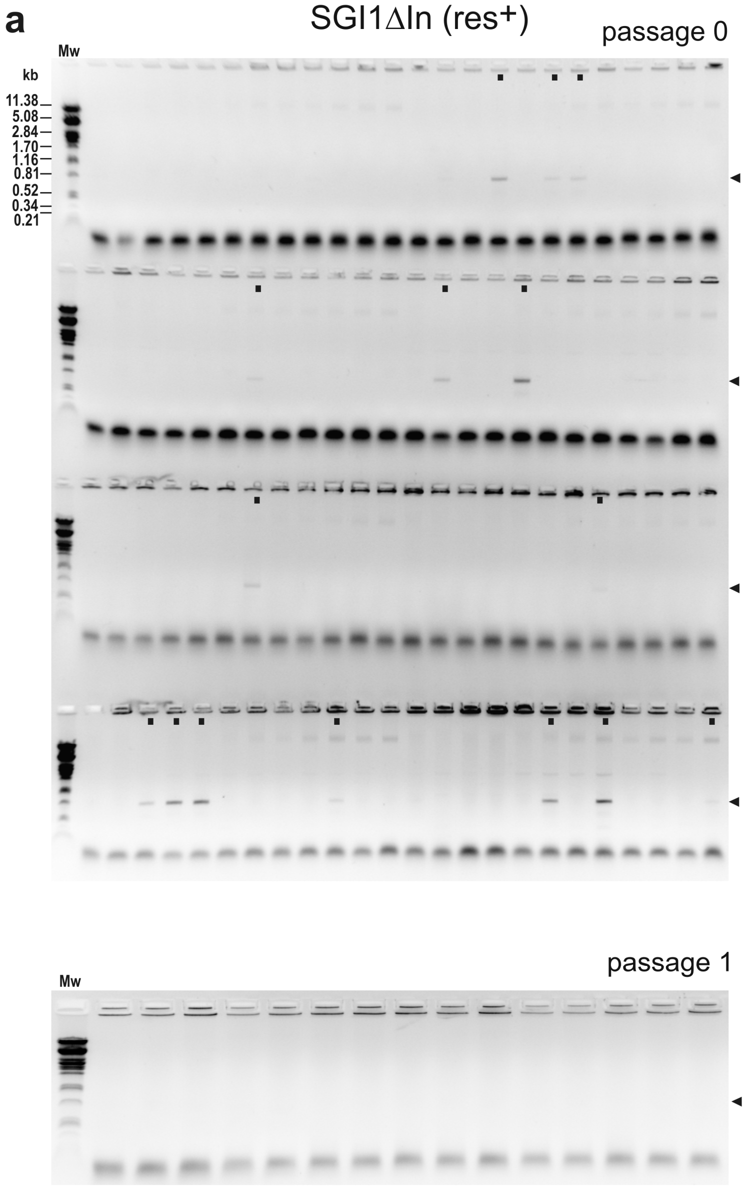

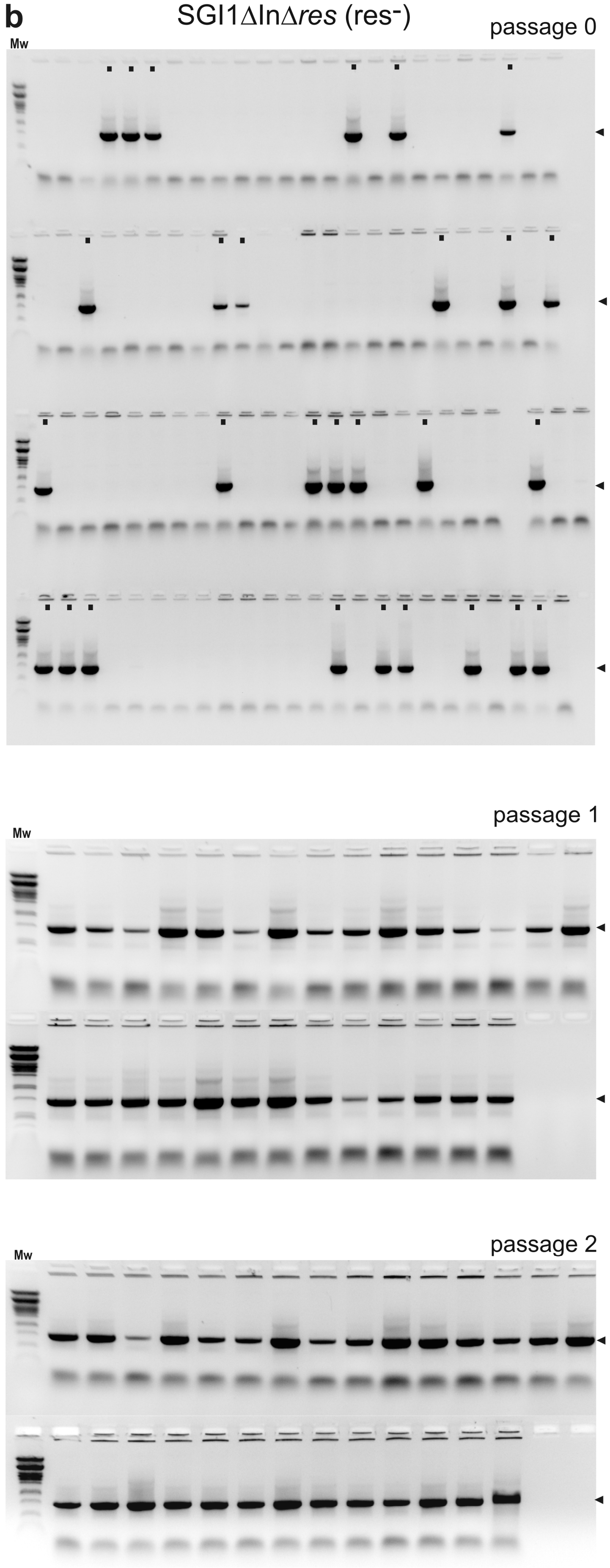

Suppl. Figure S6. Stability assay of SGI1 multimers in the presence of active (a) and inactive (b) resolution system. Upper panels show the DRL-DRR junction specific PCRs for 96 independent R55 free transconjugants (passage 0). Filled arrowheads point to the 800-bp amplicons of DRL-DRR junction, DRL-DRR^{+} clones marked with dots were passaged and tested again by the same PCR. (a) Passaging of 15 SGI1ΔIn multimer-containing clones with active resolution system (res^{+}). (b) Passaging of 28 SGI1ΔIn*Δres* multimers with inactive resolution system (res^{-}). The order of samples corresponds to those of dotted samples in Passage 0. Mw molecular-weight marker (λDNA digested with *Pst*I).

a

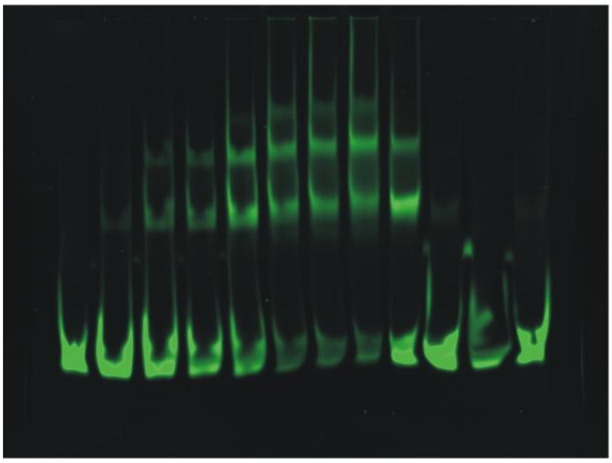

b

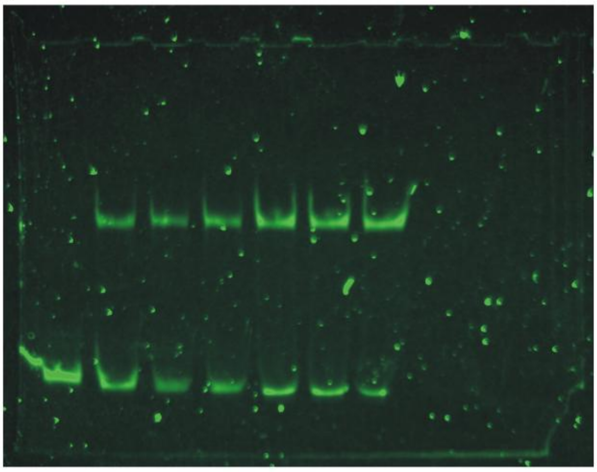

c

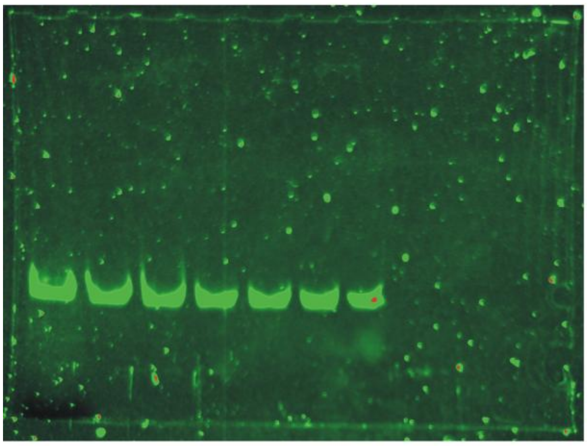

d

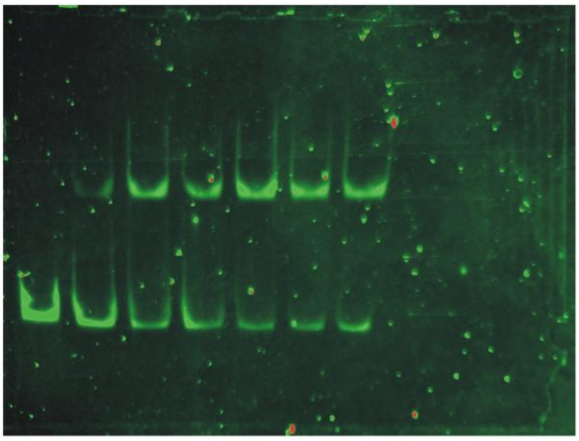

e

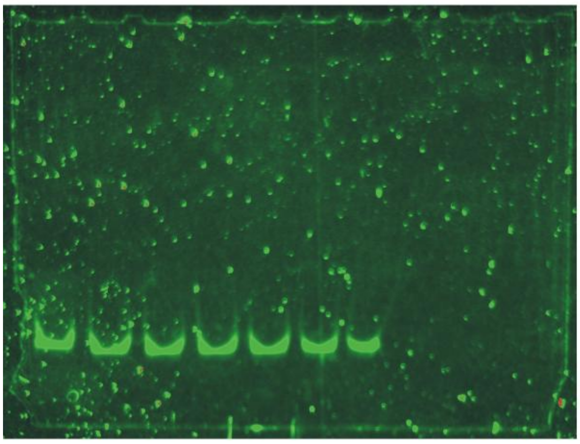

f

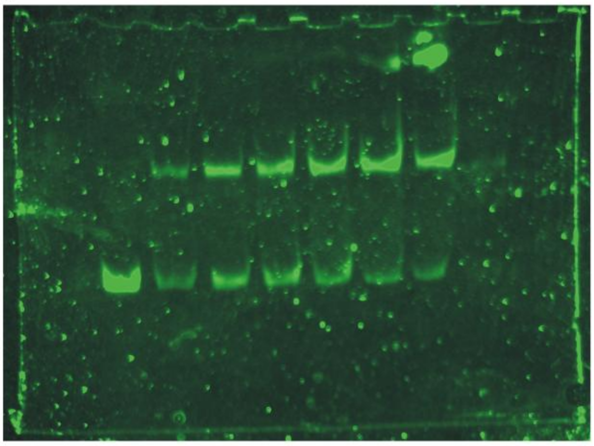

g

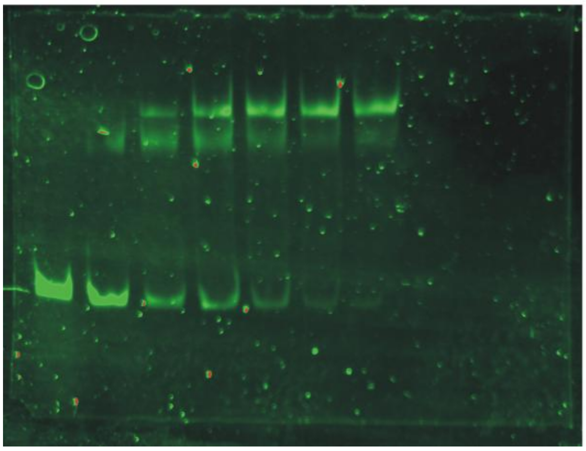

Suppl. Figure S7. Original EMSA gel images presented on Fig. 3a-g.

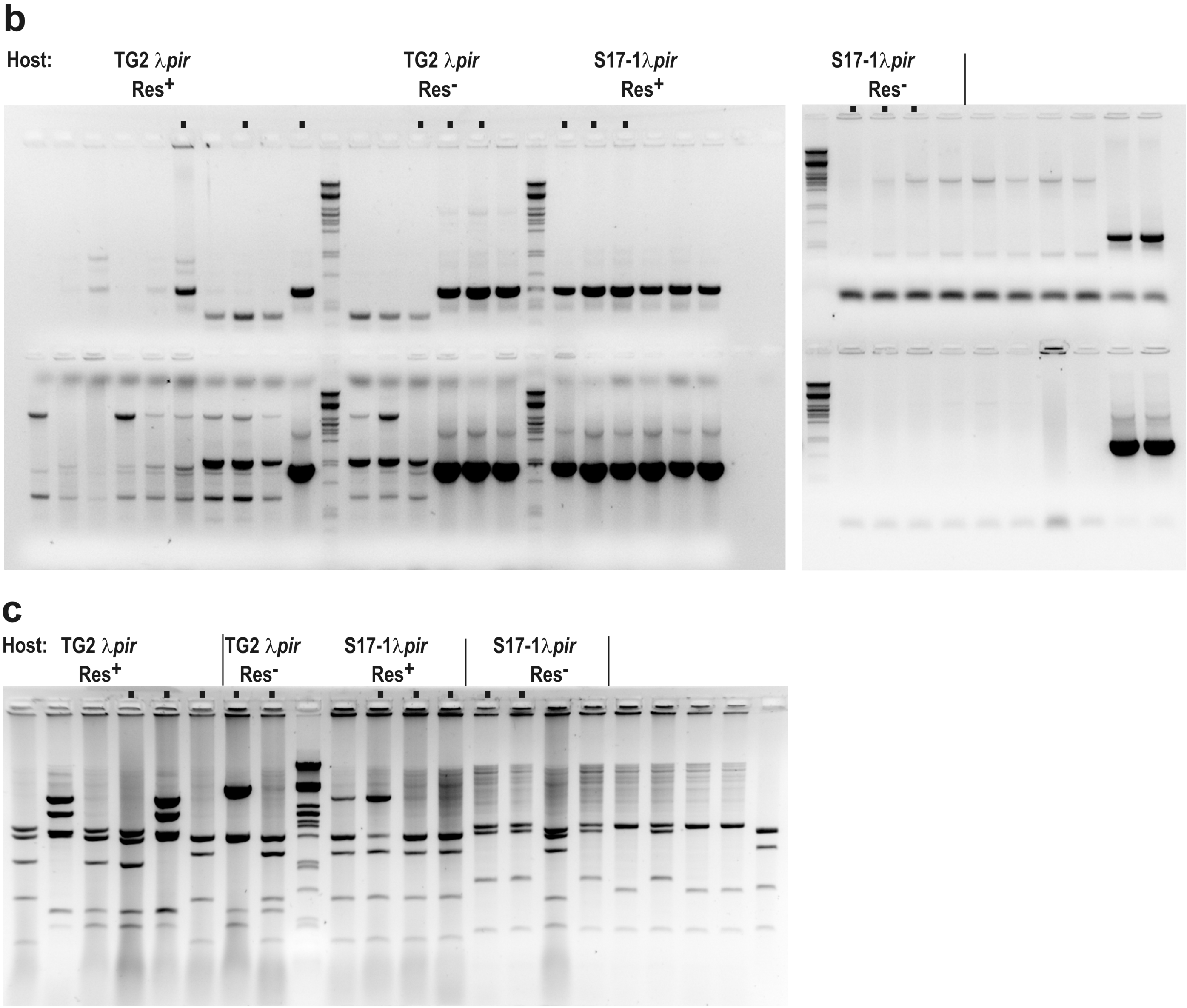

Suppl. Figure S8. Uncropped and unmodified gel images of Fig. 4b-c. Samples presented on Fig. 4 are marked with dots.

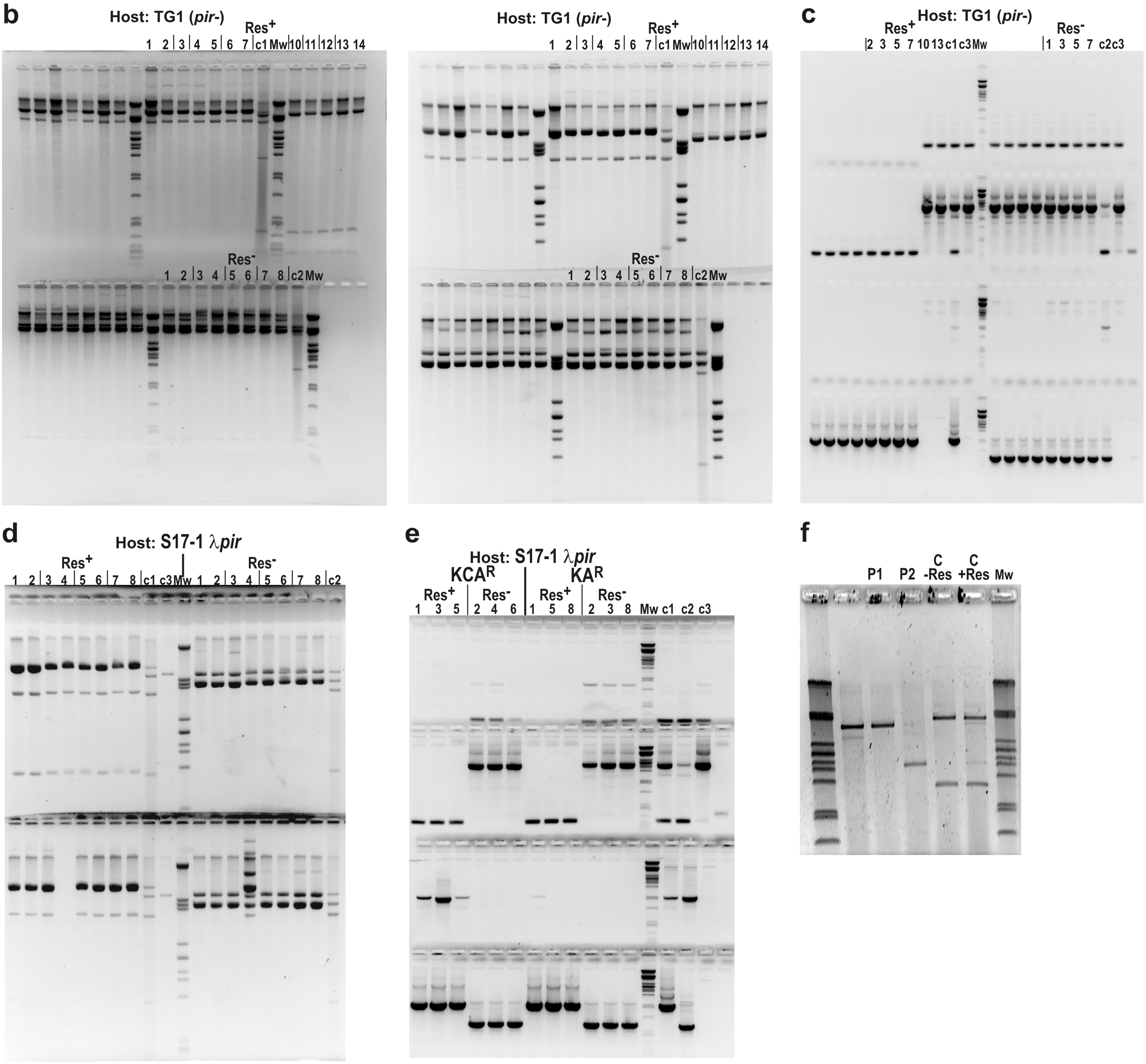

Suppl. Figure S9. Uncropped and unmodified gel images of Fig. 5b-f.

Suppl. Table S1. Relevant features of plasmids used in this study.

Name	Relevant features	References
pAVE18	pEMBL19 [1] derivative template plasmid containing the entire -S044 intergenic region (including the *res* site) located between *res* (S027) and S044 genes of SGIΔIn, Ap^R.	this work
pAVE20	pKK223-3-based producer plasmid (ColE1) expressing Res protein under the control of promoter P_{tac}, Ap^R.	this work
pAVE22	pET16b-based producer plasmid (ColE1) expressing N-terminally 10×His-tagged Res protein under the control of promoter P_{T7}, Ap^R.	this work
pAVE24	pEMBL19 derivative template plasmid containing the 28-bp *res* subsite I (boxI), Ap^R.	this work
pAVE26	pEMBL19 derivative template plasmid containing the 25-bp *res* subsite III (boxIII), Ap^R.	this work
pAVE27	pEMBL19 derivative template plasmid containing the 30-bp *res* subsite II (boxII), Ap^R.	this work
pAVE28	pEMBL19 derivative template plasmid containing the first 13-bp of *res* subsite I (half of boxI), Ap^R.	this work
pAVE30	pEMBL19 derivative template plasmid containing the 28-bp boxI, the 22 bp spacer between boxI-II and the 30-bp boxII (boxI-II), Ap^R.	this work
pAVE33	pEMBL19 derivative template plasmid containing the 30-bp boxII and the 25-bp boxIII separated by the 2-bp spacer (boxII-III), Ap^R.	this work
pCP20	Thermo-inducible FLP recombinase expression (λ pR::FLP) plasmid with temperature-sensitive pSC101 replication system, λ cI857, Ap^R,Cm^R.	[2]
pEMBL19	pMB1-based cloning vector, Ap^R.	[1]
pET16b+	ColE1-based expression vector, Ap^R.	(Novagen)
pGMY1	pSG76-CS [3] derivative (R6Kγ) template plasmid containing *aadA1* gene, Sm^R,Sp^R,Cm^S.	this work
pJKI1088	pJKI691 (p15A) derivative plasmid containing the 113-bp full *res* site (boxI-III) of SGIΔIn, Km^R.	this work
pJKI1092	pSG76-C (R6Kγ) derivative plasmid containing the 113-bp full *res* site (boxI-III) of SGIΔIn, Cm^R.	this work
pJKI1099	Cointegrate of pJKI1088 and pJKI1092 formed via recombination between the full *res* sites of parental plasmids, Km^R,Cm^R.	this work
pKD3	R6Kγ-based template plasmid, Cm^R,Ap^R.	[4]
pKD46	L-arabinose-inducible λ Red recombinase producer plasmid with temperature-sensitive pSC101 replication system, Ap^R.	[4]
pKK223-3	ColE1-based expression vector with IPTG-inducible P_{tac} promoter, Ap^R.	[5]
pMSZ934	Ap^R, Km^R mobilizable derivative of the I-SceI producer plasmid pSTKST [3] with temperature-sensitive pSC101 replication system, Tc^R, P_{tet}::SCEI, $oriT_{RK2}$.	[6]
pSG76-CS	R6Kγ based template plasmid, Cm^R.	[3]
R55	IncC Type2, *tra*+ conjugative plasmid, Cm^R,Flo^R,Sul^R,Ap^R,Km^R,Gm^R.	[7]

Suppl. Table S2. Oligonucleotide primers used.

Name	Sequence (5’→3’)[a]	References
attsgi1for	gctctagagcggccgcatggaaggcggcttcctggc	[8]
attsgifor2	caacatggaaggcggcttcctg	[9]
attsgirev2	tgacagtcagaatgcggcttcg	[9]
attsgirev3	ctggagaaaatccgccccagc	[9]
boxI_forE	aattctgttcaacaaaccaccattttcttaacactgca	this work
boxI_revP	gtgttaagaaaatggtggtttgttgaacag	this work
boxIIb_forE	aattctggaatttaatctatatacatgttaaaatactgca	this work
boxIIb_revP	gtattttaacatgtatatagattaaattccag	this work
boxIII_forE	aattctgtataaaattctatgttaaaaaaactgca	this work
boxIII_revP	gttttttaacatagaattttatacag	this work
boxIhalf_forE1	aattctgttcaacaaacctgca	this work
boxIhalf_revP1	ggtttgttgaacag	this work
boxI-II_forE1	aattctgttcaacaaaccaccattttcttaacagttcataacttagcaagttgat	this work
boxI-II_forE2	tggaatttaatctatatacatgttaaaatactgca	this work
boxI-II_revP1	gtattttaacatgtatatagattaaattccaatcaacttgctaagttatga	this work
boxI-II_revP2	actgttaagaaaatggtggtttgttgaacag	this work
boxII_III_forE	aattctggaatttaatctatatacatgttaaaatacttgtataaaattctatgttaaaaaaactgca	this work
boxII_III_revP	gttttttaacatagaattttatacaagtattttaacatgtatatagattaaattccag	this work
cat3.2	atgaattccaacagtactgcgatgagtggc	this work
catNco	tttcaccatgggcaaatattatac	this work
EMSA_res_F_5’FAM	cttaatacctccataggattaac	this work
EMSA_res_R_5’FAM	gtacgttttttaacatagaatt	this work
EMSA_for2_5’FAM	catgtacgttttttaacatagaattttatac	this work
EMSA_rev2_5’FAM	attttcttgttcaacaaaccacc	this work
delIn104AB	acaaaccaccattttcttaacagttcataacttagcaagttgattggaatttaatctatatacatgttaaaatacttgTCAACAGGT TGAACTGCGGATC	this work
delIn104C	tgaacgtcagaagccgactgcactatagcagcggaggggttggatccatcGATTTAGGTGACACTATAGAAT AC	this work
delIn104seqfor	ggtctagcggcattcttcccac	this work
d043seqrev	ggacggtttgttttcgcttgac	this work
FRTfor	atcgatgaattgatccgaagttcc	this work
LJ2	agctgcagcggccgcaagttttactctgtcttccag	[8]
LJ3	aacctaaccataagagaacttcc	[9]
ParaB	aattagagctctaaggaggttataacatatgtctagacagctgcggccgcaattgctcgaggatcctgcagc	this work
ParaBrev	aattgctgcaggatcctcgagcaattgcggccgcagctgtctagacatatgttataacctccttagagctct	this work
pBRBgl	ttaccatctggccccagtgctgc	[10]
pKKfor	ttgaattctatggctgtgcaggtcgtaaatc	this work
promcatfor	aatctcgagaacttttggcgaaaatgagacg	[11]
promcatrev	attgtcgacaagctttttagcttccttagctcctgaaaatc	[11]
pucfor24_5’FAM	FAM-cgccagggttttcccagtcacgac	[12]
pucrev25_5’FAM	FAM-atttcacacaggaaacagctatgac	[12]
pucfor24	cgccagggttttcccagtcacgac	[13]
pucrev25	atttcacacaggaaacagctatgac	[13]
resforE	aagaattcatgtacgttttttaacatagaattttatacaag	this work
resforSBg	aaagtcgacagatctatgtacgttttttaacatagaattttatacaag	this work
resrevPB	aactgcagggatccttaatacctccataggattaactatatatagaac	this work
resrevSBsp	aagtcgactccggacttaatacctccataggattaactatatatagaac	this work
resrev2PB	aactgcagggatccgcttttattttcttgttcaacaaaccacc	this work
resrev2SBsp	aaaagtcgactccggagcttttattttcttgttcaacaaaccacc	this work
resNdefor	aacgcatatgaatcaaaaaattggctacgctaggg	this work
resBamrev	aaggatccttaatcccctctagaattttgtacactc	this work
RJ2	agctgcagcggccgctcgaagaggtagagcag	[8]
RJ5	gatcagggagaattcgagtagag	[9]
rrnBrev2	aatctagagtcgacaaacaaaagagtttgtagaaacgc	[14]
SmRforSmP	cgctgcagcccggggttgccgggtgacgcac	this work
SmRrevSmP	aactgcagcccgggtcggcttgaacgaattgttagac	this work
Sm/Spinsfor	aaaagcgccttttgggcggtgtttggatgctcaactaacttactatcggcCCCGGGGTTGCCGGGTGACGCAC	this work
Sm/Spinsrev	gctctgctcagttagaatattttggatatacatctccttttgttaagtgaCCCGGGTCGGCTTGAACGAATTG	this work
027delfor	ccaattttatacaataaagtctgtgtagtatgacagggtttaagcacttaCATATGAATATCCTCCTTAGTTC	this work
027delrev	atacatgttaaaatacttgtataaaattctatgttaaaaaaacgtacatgGTGTAGGCTGGAGCTGCTT	[12]

[a] Uppercase indicates the 3’ part of KO oligos that anneals to template plasmids pKD3 or pSG76-CS. Restriction sites are underlined.

Suppl. Table S3. List of bacterial strains

E. coli strains	Genotypes	References
TG1	*E. coli* K-12 derivative, *supE hsdΔ5 thiΔ*(*lac-proAB*) F’[*traD36 proAB*$^+$ *lacIq lacZΔM15*].	15
TG2/R55	TG2 strain [16] containing the conjugative IncC plasmid R55, Tc^R,Ap^R,Cm^R,Flo^R,Km^R,Gm^R,Sul^R.	17
TG90	TG1 derivative, *pcn B80 zad*::Tn*10*, Tc^R.	18
BL21 (DE3)	*E. coli* B derivative, *hsdS gal (λcIts857 ind*1 *Sam*7 *nin*5 *lac*UV5-T7 gene 1).	19
TG1Nal::SGI1-C (wt)	SGI1-C transconjugant TG1Nal strain [8] carrying a single copy of chromosomally integrated SGI1-C at *attB* site (*trmE*), Nal^R,Sm^R,Sp^R,Sul^R.	14
TG1Nal::SGI1ΔIn	TG1Nal::SGI1-C derivative carrying a single copy of chromosomally integrated, In104-deleted SGI1-C (i. e. *res* site is restored) with *aadA1* gene inserted downstream of S023, Nal^R,Sm^R,Sp^R,Sul^R.	this work
TG1Nal::SGI1ΔInΔ*res*	TG1Nal::SGI1ΔIn derivative carrying a single copy of chromosomally integrated SGI1ΔIn with Δ*res* deletion (i. e. the *res* [S027] gene is removed), Nal^R,Sm^R,Sp^R,Sul^R.	this work
S17-1 λ*pir*	λ lysogen derivative of S17-1 (*pro thi recA hsdR* (r^- m^+) [Ω RP4-2-Tc::Mu-Km::Tn*7*]) expressing Π protein from *pir* gene of plasmid R6K, Tp^R,Sm^R,Km^S.	20
TG2 λ*pir*	TG2 derivative [16] obtained by lysogenization with λ*pir* phage isolated from S17-1 λ*pir* strain.	21

Suppl. Table S4. List of EMSA probes

**Sign**	***res* site region included**	**Primers used for cloning the *res* site region into template plasmids**	**Template plasmid**	**Primers used for amplification of labelled probe for EMSA**	**Total length (bp)**
-	S027-S044 intergenic region (including the restored full *res* site)	resforSBg – resrevSBsp	pAVE18	EMSA_res_F_5’FAM and EMSA_res_R_5’FAM	207
A	full *res* site (boxI-III, 1-119 bp of S027-S044 intergenic region)[a]	resforSBg – resrevSBsp	pAVE18	EMSA_for2_5’FAM and EMSA_rev2_5’FAM	122
B	*res* boxI (85-112 bp of S027-S044 intergenic region)	boxI_forE – boxI_revP	pAVE24	pucfor24_5′FAM and pucrev25_5′FAM	133
C	*res* boxII (33-62 bp of S027-S044 intergenic region)	boxIIb_forE – boxIIb_revP	pAVE27	pucfor24_5′FAM and pucrev25_5′FAM	135
D	*res* boxIII (6-30 bp of S027-S044 intergenic region)	boxIII_forE – boxIII_revP	pAVE26	pucfor24_5′FAM and pucrev25_5′FAM	130
E	half of *res* boxI (101-112 bp of S027-S044 intergenic region)	boxIhalf_forE1 – boxIhalf_revP1	pAVE28	pucfor24_5′FAM and pucrev25_5′FAM	117
F	*res* boxI-II (32-112 bp of S027-S044 intergenic region, including boxI, spacer of boxI-II, and boxII)	boxI_II_forE1 – boxI_II_revP1	pAVE30	pucfor24_5′FAM and pucrev25_5′FAM	185
G	*res* boxII-III (6-62 bp of S027-S044 intergenic region, including boxII, spacer of boxII-III, and boxIII)	boxII_III_forE – boxII_III_revP	pAVE33	pucfor24_5′FAM and pucrev25_5′FAM	162

[a] Numbering starts from G base preceding the start codon of *res* gene in SGI1ΔIn.

Supplementary Text S1.

SGI1 fragments were amplified using the genomic DNA of TG1Nal::SGI1-C or TG1Nal::SGI1ΔIn strains as template DNA unless otherwise specified.

pAVE18: The 27558-27766 bp intergenic region of SGI1ΔIn, including the restored *res* site (boxI-III) was amplified with primers resforSBg and resrevSBsp. The amplicon was digested with *Sal*I and cloned into the *Sal*I-digested vector pEMBL19 [1].

pAVE20: The *res* gene (S027) of SGI1 was amplified with primers resNdefor and resBamrev. The amplicon was digested with *Nde*I-*Bam*HI and ligated into *Nde*I-*Bam*HI-digested pJKI397, a pKK223-3 [5] derivative expression vector that is analogous to pJKI132 [22] (the *res* gene was substituted for the originally present IS*30* transposase gene).

pAVE22: The *res* gene (S027) of SGI1 was amplified with primers resNdefor and resBamrev. The amplicon was digested with *Nde*I-*Bam*HI and ligated into the *Nde*I-*Bam*HI-digested pET16b (Novagen).

pAVE24: The boxI_forE and boxI-revP oligonucleotides, representing the 28-bp predicted *res* subsite I (boxI), were annealed and ligated into the *Eco*RI-*Pst*I site of pEMBL19.

pAVE26: The boxIII_forE and boxIII-revP oligonucleotides, representing the 25-bp predicted *res* subsite III (boxIII), were annealed and ligated into the *Eco*RI-*Pst*I site of pEMBL19.

pAVE27: The boxIIb_forE and boxIIb-revP oligonucleotides, representing the 30-bp predicted *res* subsite II (boxII), were annealed and ligated into the *Eco*RI-*Pst*I site of pEMBL19.

pAVE28: The boxIhalf_forE1 and boxIhalf-revP1 oligonucleotides, representing the 12-bp left arm of the putative single Res binding site of the predicted *res* subsite I (boxI) (half of the inverted repeat without the central 4-bp spacer), were annealed and ligated into the *Eco*RI-*Pst*I site of pEMBL19.

pAVE30: The boxI-II_forE1, boxI-II_forE2, boxI-II_revP1 and boxI-II_revP2 oligonucleotides, representing the predicted *res* subsites I and II with the spacer, i. e. the 28-bp boxI, the 22-bp spacer between boxI and boxII, and the 30-bp boxII, were annealed and ligated into the *Eco*RI-*Pst*I site of pEMBL19.

pAVE33:The boxII-III_forE and boxII-III-revP oligonucleotides, representing the predicted *res* subsites II and III, i. e. the 28-bp boxI, the 2-bp spacer between boxII and boxIII and the 25-bp boxIII, were annealed and ligated into the *Eco*RI-*Pst*I site of pEMBL19.

pGMY1: The 1152 bp fragment of pHP45Ω [23] carrying the Sm^R/Sp^R gene (*aadA1*) was amplified with primers SmRforSmP and SmRrevSmP. The amplicon was digested with *Pst*I and cloned into the *Pst*I-digested pJKI332 [6], replacing its Km^R cassette. The two *Xba*I sites in the multicloning site were eliminated in the resulting plasmid pJKI630 by *Xba*I-digestion followed by self-ligation after blunting the sticky ends with Klenow polymerase, which led to pJKI733. The Sm^R/Sp^R cassette of pJKI733 was cut out by *Not*I-*Sal*I digestion and ligated into the appropriately digested pSG76-CS [3], leading to the replacement of its original Cm^R gene to Sm^R/Sp^R.

pJKI1088: The 131-bp fragment containing the predicted full *res* site (boxI-III) was amplified from SGI1ΔIn with primers resforE and resrev2PB. The amplicon was digested with *Eco*RI-*Pst*I and ligated into the *Eco*RI-*Pst*I-digested pJKI691.

pJKI1092: The 145-bp *Sal*I fragment containing the predicted full *res* site (boxI-III) was cut out from pJKI1090 and ligated into the *Sal*I site of pSG76-C.

pJKI1099: Cointegrate of pJKI1088 and pJKI1092 formed via recombination between the full *res* sites of the parental plasmids.

Supplementary references

1. Dente, L., Cesareni, G. & Cortese, R. pEMBL: A new family of single stranded plasmids. *Nucleic Acids Res.* **11**, 1645–1655 (1983).
2. Cherepanov, P. P. & Wackernagel, W. Gene disruption in *Escherichia coli*: TcR and KmR cassettes with the option of Flp-catalyzed excision of the antibiotic-resistance determinant. *Gene* **158**, 9–14 (1995).
3. Kolisnychenko, V. *et al.* Engineering a reduced *Escherichia coli* genome. *Genome Res.* **12**, 640–7 (2002).
4. Datsenko, K. A. & Wanner, B. L. One-step inactivation of chromosomal genes in *Escherichia coli* K-12 using PCR products. *Proc. Natl. Acad. Sci.* **97**, 6640–6645 (2000).
5. Brosius, J. & Holy, A. Regulation of ribosomal RNA promoters with a synthetic lac operator. *Proc. Natl. Acad. Sci. U. S. A.* **81**, 6929–6933 (1984).
6. Kiss, J. *et al.* Identification and Characterization of oriT and Two Mobilization Genes Required for Conjugative Transfer of *Salmonella* Genomic Island 1. *Front. Microbiol.* **10**, 1–16 (2019).
7. Chabbert, Y. A., Scavizzi, M. R., Witchitz, J. L., Gerbaud, G. R. & Bouanchaud, D. H. Incompatibility Groups and the Classification of f- Resistance Factors. *J. Bacteriol.* **112**, 666–675 (1972).
8. Kiss, J., Nagy, B. & Olasz, F. Stability, entrapment and variant formation of *Salmonella* genomic island 1. *PLoS One* **7**, e32497 (2012).
9. Szabó, M., Murányi, G. & Kiss, J. IncC helper dependent plasmid-like replication of *Salmonella* Genomic Island 1. *Nucleic Acids Res.* **49**, 832–846 (2021).
10. Kiss, J. *et al.* Transposition and target specificity of the typical IS*30* family element IS*1655* from *Neisseria meningitidis*. *Mol. Microbiol.* **63**, 1731–1747 (2007).
11. Nagy, I., Szabó, M., Hegyi, A. & Kiss, J. Salmonella Genomic Island 1 requires a self-encoded small RNA

for mobilization. *Mol. Microbiol.* **116**, 1533–1551 (2021).
12. Murányi, G., Szabó, M., Acsai, K. & Kiss, J. Two birds with one stone: SGI1 can stabilize itself and expel the IncC helper by hijacking the plasmid *parABS* system. *Nucleic Acids Res.* **52**, 2498–2518 (2024).
13. Szabó, M., Kiss, J., Nagy, Z., Chandler, M. & Olasz, F. Sub-terminal Sequences Modulating IS*30* Transposition in Vivo and in Vitro. *J. Mol. Biol.* **375**, (2008).
14. Kiss, J. *et al.* The master regulator of IncA/C plasmids is recognized by the *Salmonella* Genomic island SGI1 as a signal for excision and conjugal transfer. *Nucleic Acids Res.* **43**, 8735–8745 (2015).
15. Gibson, T. J. Studies on the Epstein-Barr virus genome. Thesis. (Cambridge. UK, 1984).
16. Sambrook, J., Fritsch, E. F. & Maniatis, T. *Molecular Cloning: A Laboratory Manual. Cold Spring Harbor Laboratory Press, Cold Spring Harbor, NY. New York* (1989).
17. Hegyi, A., Szabó, M., Olasz, F. & Kiss, J. Identification of oriT and a recombination hot spot in the IncA/C plasmid backbone. *Sci. Rep.* **7**, 10595 (2017).
18. Gonzy-Treboul, G., Karmazyn-Campelli, C. & Stragier, P. Developmental regulation of transcription of the *Bacillus subtilis ftsAZ* operon. *J. Mol. Biol.* **224**, 967–979 (1992).
19. Studier, F. W. & Moffatt, B. A. Use of bacteriophage T7 RNA polymerase to direct selective high-level expression of cloned genes. *J. Mol. Biol.* **189**, 113–130 (1986).
20. Simon, R., Priefer, U. & Pühler, A. A Broad Host Range Mobilization System for In Vivo Genetic Engineering: Transposon Mutagenesis in Gram Negative Bacteria. *Bio/Technology* **1**, 784–791 (1983).
21. Veress, A. *et al.* Abundance of mobile genetic elements in an *Acinetobacter lwoffii* strain isolated from Transylvanian honey sample. *Sci. Rep.* **10**, 1–14 (2020).
22. Farkas, T., Kiss, J. & Olasz, F. The construction and characterization of an effective transpositional system based on IS*30*. *FEBS Lett.* **390**, 53–58 (1996).
23. Prentki, P. & Krisch, H. M. In vitro insertional mutagenesis with a selectable DNA fragment. *Gene* **29**, 303–313 (1984).
